# Supplementary material for: Allele-specific gene-editing approach for vision loss restoration in RHO-associated retinitis pigmentosa
Source: eLife. 2023 Jun 5;12:e84065. doi: 10.7554/eLife.84065 (PMC10279453; doi:10.7554/eLife.84065)
Supplement: Supplementary file 1. — (a) Results of TA cloning and Sanger sequencing in 293T cells. (b) A comprehensive summary of mouse experiments. (c) Results of TA cloning and Sanger sequencing in AAV-based SaCas9/17-Sg2-treated Mut-Rhowt/hum retinas. (d) Off-target sites of 17-Sg2 obtained from Benchling (https://www.benchling.com/). (e) Off-target sites of 17-Sg2 obtained from Cas-OFFinder (http://www.rgenome.net/cas-offinder) and NGS results. (f) Off-target sites of 17-Sg2 obtained from Cas-OFFinder (http://www.rgenome.net/cas-offinder) and NGS results. (g) The number of off-target sites of 17-Sg2 obtained from Cas-OFFinder (http://www.rgenome.net/cas-offinder). (h) Whole-genome sequencing results of off-target activity for SaCas9/17Sg-2. (i) List of primers. [file elife-84065-supp1.docx]

**Supplementary file 1**

**Allele-specific gene editing approach for vision loss restoration in**

***RHO*-associated Retinitis Pigmentosa**

Xiaozhen Liu, Jing Qiao, Ruixuan Jia, Fan Zhang, Xiang Meng, Liping Yang

**Table of content**

Supplementary file 1a-1i

| **Supplementary file 1a.** **Results of TA cloning and Sanger Sequencing in 293T cells.** | | |
| --- | --- | --- |
| **RHO17-Sg1** | | |
| **Nucleotide changes** | **Amino acid changes** | **Number of clones** |
| c.41_50del | p.S14Wfs*31 | 1 |
| c.43_44del | p.N15Cfs*18 | 1 |
| c.44_45del | p.N15Sfs*18 | 1 |
| c.44_45insA | p.N15Kfs*19 | 1 |
| c.44_45ins341bp | p.A16Cfs*9 | 1 |
| c.44_45ins156bp | p.A16Qfs*41 | 1 |
| c.44_45ins45bp | insertion of more than 2 AA | 1 |
| Total | | 7 |
| T>C | p.T17 | 5 |
| **RHO17-Sg2** | | |
| **Nucleotide changes** | **Amino acid changes** | **Number of clones** |
| c.24_50del | deletion of more than 2 AA | 1 |
| c.43_46del | p.N15Rfs*32 | 1 |
| c.45_46del | p.A16Dfs*17 | 2 |
| c.46del | p.A16Rfs*32 | 2 |
| c.47_51del | p.A16Gfs*16 | 1 |
| c.47_52del | deletion of 1 or 2 AA | 1 |
| c.47_57del | p.A16Gfs*14 | 1 |
| c.49_62del | p.T17Qfs*12 | 1 |
| c.46_47 ins75bp | p.A16Efs*16 | 1 |
| c.37_47delinsA | p.F13Rfs*32 | 1 |
| c.43_46del4bpinsCA | p.N15Hfs*18 | 1 |
| c.47_51delins19bp | deletion of 1 or 2 AA | 1 |
| c.50delT ins 44bp | p.T17Nfs*31 | 1 |
| Total | | 15 |

**Supplementary file 1b. A comprehensive summary of mouse experiments.**

| **Step 1** | | | | | | | | | | | | | | |
| --- | --- | --- | --- | --- | --- | --- | --- | --- | --- | --- | --- | --- | --- | --- |
| Mouse strain | Treatment age | Eye | Treatment | Injection volume(totally) | AAV dose(vg/eye) | T7E1 assay | TA and Sanger sequencing | Hi-Tom sequencing | ERG | Cryosection | IF | HE | RT-PCR | WB |
| *Rho ^wt/wt^* | P2m | Both(*n*=4) | AAV2/8-EFS-EGFP | 1μl | 1×10^9^ | n/a | n/a | n/a | n/a | 1m p.i.(*n*=2) | 1m p.i.(*n*=2) | n/a | n/a | n/a |
|  |  |  |  |  |  |  |  |  |  | 11m p.i.(*n*=2) | 11m p.i.(*n*=2) |  |  |  |
| *Rho ^wt/wt^* | P2m | Both(*n*=4) | AAV2/8-EFS-EGFP | 1μl | 3×10^9^ | n/a | n/a | n/a | n/a | 1m p.i.(*n*=2) | 1m p.i.(*n*=2) | n/a | n/a | n/a |
|  |  |  |  |  |  |  |  |  |  | 11m p.i.(*n*=2) | 11m p.i.(*n*=2) |  |  |  |
| **Step 2** | | | | | | | | | | | | | | |
| Mouse strain | Treatment age | Eye | Treatment | Injection volume (totally) | AAV dose(vg/eye) | T7E1 assay | TA&Sanger sequencing | Hi-Tom sequencing | ERG | Cryosection | IF | HE | RT-PCR | WB |
| *Rho ^wt/hum^* | P1.5-1.8m | Both(*n*=4) | AAV2/8-EFS-EGFP and AAV2/8-EFS-SaCas9-U6-17-Sg2 | 1μl | 5×10^8^ each | 3m *p.i.*(*n*=2) | 3m *p.i.*(*n*=2) | 3m *p.i.*(*n*=2) | n/a | n/a | n/a | n/a | 3m *p.i.*(*n*=2) | n/a |
| *Rho ^wt/hum^* | P1.5-1.8m | Both(*n*=8) |  | 1μl | 1×10^9^ each | 3m *p.i.*(*n*=3) | 3m *p.i.*(*n*=3) | 3m *p.i.*(*n*=3) | n/a | n/a | n/a | n/a | 3m *p.i.*(*n*=2) | 3m *p.i.*(*n*=3) |
| *Rho ^wt/hum^* | P1.5-1.8m | Both(*n*=10) |  | 2μl | 3×10^9^ each | 3m *p.i.*(*n*=3) | 3m *p.i.*(*n*=3) | 3m *p.i.*(*n*=3) | n/a | n/a | n/a | n/a | 3m *p.i.*(*n*=3) | 3m *p.i.*(*n*=4) |
| Mut-*Rho ^wt/hum^* | P1.5-1.8m | Both(*n*=3) |  | 1μl | 5×10^8^ each | 3m *p.i.*(*n*=3) | 3m *p.i.*(*n*=1) | 3m *p.i.*(*n*=1) | n/a | n/a | n/a | n/a | n/a | n/a |
| Mut-*Rho ^wt/hum^* | P1.5-1.8m | Both(*n*=4) |  | 1μl | 1×10^9^ each | 3m *p.i.*(*n*=4) | 3m *p.i.*(*n*=4) | 3m *p.i.*(*n*=4) | n/a | n/a | n/a | n/a | n/a | n/a |
| Mut-*Rho ^wt/hum^* | P1.5-1.8m | Both(*n*=5) |  | 2μl | 3×10^9^ each | 3m *p.i.*(*n*=5) | 3m *p.i.*(*n*=5) | 3m *p.i.*(*n*=5) | n/a | n/a | n/a | n/a | n/a | n/a |
| Mut-*Rho ^hum/hum^* | P1.5-1.8m | Both(*n*=2) |  | 1μl | 1×10^9^ each | 3m *p.i.*(*n*=2) | n/a | n/a | n/a | n/a | n/a | n/a | n/a | n/a |
| Mut-*Rho ^hum/hum^* | P1.5-1.8m | Both(*n*=2) |  | 2μl | 3×10^9^ each | 3m *p.i.*(*n*=2) | n/a | n/a | n/a | n/a | n/a | n/a | n/a | n/a |
| **Step 3** | | | | | | | | | | | | | | |
| Mouse strain | Treatment age | Eye | Treatment | Injection volume (totally) | AAV dose(vg/eye) | T7E1 assay | TA&Sanger sequencing | Hi-Tom sequencing | ERG | Cryosection | IF | HE | RT-PCR | WB |
| *Rho ^hum/m-hum^* | P1.5-1.8m | Both(*n*=4) | AAV2/8-EFS-EGFP and AAV2/8-EFS-SaCas9-U6-17-Sg2 | 1μl | 1×10^9^ each | 3m *p.i.*(*n*=4) | 3m *p.i.*(*n*=4) | 3m *p.i.*(*n*=4) | n/a | n/a | n/a | n/a | 3m *p.i.*(*n*=3) | n/a |
| *Rho ^hum/m-hum^* | P1.5-1.8m | Both(*n*=4) |  | 2μl | 3×10^9^ each | 3m *p.i.*(*n*=4) | 3m *p.i.*(*n*=4) | 3m *p.i.*(*n*=4) | n/a | n/a | n/a | n/a | 3m *p.i.*(*n*=4) | n/a |
| **Step 4** | | | | | | | | | | | | | | |
| Mouse strain | Treatment age | Eye | Treatment | Injection volume (totally) | AAV dose(vg/eye) | T7E1 assay | TA&Sanger sequencing | Hi-Tom sequencing | ERG | Cryosection | IF | HE | RT-PCR | WB |
| Mut-*Rho ^wt/hum^* | P1.5-1.8m | Both(*n*=5) | AAV2/8-EFS-EGFP and AAV2/8-EFS-SaCas9-U6-CTRL | 1μl | 1×10^9^ each | n/a | n/a | n/a | 3m *p.i*.(*n*=5) | 4m *p.i.*(*n*=3) | 4m *p.i.*(*n*=3) | 4m *p.i.*(*n*=3) | n/a | n/a |
| Mut-*Rho ^wt/hum^* | P1.5-1.8m | Both(*n*=5) |  | 2μl | 3×10^9^ each | n/a | n/a | n/a | 3m *p.i*.(*n*=5) | 4m *p.i.*(*n*=3) | 4m *p.i.*(*n*=3) | 4m *p.i.*(*n*=3) | n/a | n/a |
| Mut-*Rho ^wt/hum^* | P1.5-1.8m | Both(*n*=7) | AAV2/8-EFS-EGFP and AAV2/8-EFS-SaCas9-U6-17-Sg2 | 1μl | 1×10^9^ each | n/a | n/a | n/a | 3m *p.i*.(*n*=7) | 4m *p.i.*(*n*=3) | 4m *p.i.*(*n*=3) | 4m *p.i.*(*n*=3) | n/a | n/a |
| Mut-*Rho ^wt/hum^* | P1.5-1.8m | Both(*n*=9) |  | 2μl | 3×10^9^ each | n/a | n/a | n/a | 3m *p.i*.(*n*=9) | 4m *p.i.*(*n*=3) | 4m *p.i.*(*n*=3) | 4m *p.i.*(*n*=3) | n/a | n/a |
|  |  |  |  |  |  |  |  |  |  | 9m *p.i.*(n=3) | 9m *p.i.*(n=3) | 9m *p.i.*(n=3) | n/a | n/a |

*n*= the number of the treated mouse.

**Supplementary file 1c. Results of TA cloning & Sanger sequencing in AAV-based SaCas9/17-Sg2-treated Mut-*Rho^wt/hum^* Retinas.**

| Nucleotide changes | Amino acid changes | #M1-GFP+ | #M2-GFP+ | #M3-GFP+ | #M4-GFP+ | #M5-GFP+ | #M6-GFP+ | #M7-all | #M8-all | #M9-all | #M10-all | Total |
| --- | --- | --- | --- | --- | --- | --- | --- | --- | --- | --- | --- | --- |
| c.29_360+104del436bp | - |  |  |  |  |  |  |  |  |  | 1 | 1 |
| c.38_46del9bp | deletion of more than 2 AA |  |  |  |  |  |  | 1 |  |  |  | 1 |
| c.40_47del8bp | p.S14Dfs*17 |  |  |  |  |  | 1 |  |  |  |  | 1 |
| c.41C>G | p. S14C |  |  |  |  |  |  |  |  | 1 |  | 1 |
| c.42_47del6bp | deletion of 1 or 2 AA |  |  |  |  |  |  |  |  | 1 |  | 1 |
| c.43_46del4bp | p.N15Rfs*32 |  |  |  |  |  | 2 |  |  |  |  | 2 |
| c.43_47del5bp | p.N15Dfs*17 |  |  |  |  | 1 |  |  |  |  |  | 1 |
| c.44_46del3bp | deletion of 1 or 2 AA |  |  |  | 1 |  | 1 |  |  |  | 1 | 3 |
| c.44_49del6bp | deletion of 1 or 2 AA |  | 1 |  |  |  |  |  |  |  |  | 1 |
| c.45T>G | p. N15K |  |  |  | 1 |  |  |  | 1 |  |  | 2 |
| c.45_45del1bp | p.N15Kfs*33 |  | 1 |  |  |  |  |  |  |  |  | 1 |
| c.45_46del2bp | p.A16Dfs*17 |  |  |  | 1 | 1 |  |  | 1 | 2 | 1 | 6 |
| c.46_46del1bp | p.A16Rfs*32 | 1 | 1 | 1 | 1 | 3 | 5 | 2 | 3 | 1 |  | 18 |
| c.47C>G | p.A16G |  |  | 1 | 2 |  |  |  | 1 | 1 |  | 5 |
| c.47C>G;c.49_51del3bp | p.A16G; deletion of 1 or 2 AA |  |  |  |  |  |  |  |  | 1 |  | 1 |
| c.47_47del1bp | p.A16Gfs*32 |  | 1 | 1 |  | 1 | 1 | 2 | 1 |  | 2 | 9 |
| c.47_48 del2bp | p.A16Dfs*17 |  |  |  |  | 1 | 1 | 1 | 1 | 1 | 1 | 6 |
| c.47_49del3bp | deletion of 1 or 2 AA |  |  |  |  | 1 |  |  |  |  |  | 1 |
| c.47_50del4bp | p.A16Gfs*31 |  | 1 | 1 |  |  |  |  |  |  |  | 2 |
| c.47_51del5bp | p.A16Gfs*16 |  |  |  |  | 1 |  |  |  |  |  | 1 |
| c.47_54del8bp | p.A16Gfs*15 |  |  |  | 1 |  |  |  |  |  |  | 1 |
| c.47_55del9bp | deletion of more than 2 AA |  |  |  |  |  |  |  | 1 |  |  | 1 |
| c.47_57del11bp | p.A16Gfs*14 |  |  |  |  |  |  |  |  | 1 |  | 1 |
| c.47_58del12bp | deletion of more than 2 AA |  |  | 1 |  |  |  |  |  |  |  | 1 |
| c.52G>A | p.G18S |  |  | 1 |  |  |  |  |  |  |  |  |
| c.1-100_c.66 del166bp | - |  |  |  | 1 |  |  |  |  |  |  | 1 |
| c.1-99_c.92 del191bp | - |  | 1 |  |  |  |  |  |  |  |  | 1 |
| C.46_47inA | p.A16Dfs*18 |  |  |  |  |  |  |  |  |  | 1 | 1 |
| C.46_47inT | p.A16Vfs*18 |  |  |  |  |  |  |  |  |  | 1 | 1 |
| C.46_47inTA | P.A16Vfs*33 |  |  |  |  |  |  |  | 1 |  |  | 1 |
| c.47_50delinsGGTCGCCCGACGCCCGG | p.A16Gfs*22 |  |  |  |  |  |  |  | 1 |  |  | 1 |
| c.48_49delins43bp | p.M17Pfs*45 |  |  |  | 1 |  |  |  |  |  |  | 1 |
| c.47_49delins82bp | p.A16Vfs*13 |  | 1 |  |  |  |  |  |  |  |  | 1 |
| c.52_53insATGG | p.G18Dfs*17 |  |  | 1 |  |  |  |  |  |  |  | 1 |
| c.28_79delins50bp | p.Y10Qfs*13 |  |  |  |  | 1 |  |  |  |  |  | 1 |
| c.46_47insG | p.A16Gfs*18 |  |  |  |  |  |  | 1 |  |  |  | 1 |
| c.46_47ins49bp | p.A16Gfs*34 |  |  |  |  |  |  | 1 |  |  |  | 1 |
| c.46_47ins55bp | p.A16Vfs*36 |  |  |  |  |  | 1 |  |  |  |  | 1 |
| c.47_48ins71bp | p.M17Efs*55 |  |  |  |  |  |  | 1 |  |  |  | 1 |
| c.47_57delins70bp | p.A16Gfs*6 |  |  |  |  |  |  | 1 |  |  |  | 1 |
| c.48_49insCG | p.M17Rfs*32 |  |  |  |  | 1 |  |  |  |  |  | 1 |
| c.50_51insGGCGAT | deletion and insertion of 1 or 2 AA | 1 |  |  |  |  |  |  |  |  |  | 1 |
| c.50_51insGCGAT | p.G18Rfs*32 |  |  |  |  |  | 1 |  |  |  |  | 1 |
| **Number of indels** | | 2 | 7 | 7 | 9 | 11 | 13 | 10 | 11 | 9 | 8 |  |
| **Number of clones** | | 60 | 60 | 72 | 59 | 57 | 63 | 64 | 60 | 60 | 60 |  |
| **Percentage of indels** | | 0.0333 | 0.1167 | 0.0972 | 0.1525 | 0.1930 | 0.2063 | 0.15625 | 0.1833 | 0.15 | 0.1333 |  |

**Supplementary file 1d. Off-target sites of 17-Sg2 obtained from Benchling (<https://www.benchling.com/>).**

Red indicates mismatches of off-target sequence compared to 17-Sg2 sequence.

|  | Sequence | PAM | Score | Gene | Chromosome | Strand | Position | Mismatches | On-target | Forward primer | Reverse primer |
| --- | --- | --- | --- | --- | --- | --- | --- | --- | --- | --- | --- |
| n/a | **TGCGTACCACACCCaTCGCAT** | **TGGAG** | **100** | **RHO(ENSG00000163914)** | **chr3** | **-1** | **129247623** | **0** | **TRUE** | n/a | n/a |
| OT1 | GACCTACCACACCCGCCACAT | CTGAG | 0.134943182 |  | chr3 | -1 | 192513775 | 5 | FALSE | AGGCCTCTGGACCTTTTTCG | TCACGCAATCTTGACTAGAGC |
| OT2 | GGCCTAACACACCCTTCGCAG | AAGAA | 0.133327096 |  | chr15 | 1 | 95244889 | 5 | FALSE | CACCAGAGGGTGTAACTTAGCG | GTAGCTTCTGTCGGCGTGAC |
| OT3 | CCCGTACCACACCCATCACTT | TAGGA | 0.130208333 |  | chr3 | -1 | 51983185 | 5 | FALSE | AAGGCTGAATGGTACTCTGTTATGT | GAAGAATGCTTGATTCACCTGGC |
| OT4 | GGCCTACCACACCCTTGGCAA | GGGGA | 0.124388966 |  | chr9 | -1 | 137665529 | 5 | FALSE | CGTTCACCCCAAGACTGCTT | CTTTCCACATGGTGTCACGG |
| OT5 | CCCGTACCACAGCCATCCCAT | CAGGG | 0.121670082 | NAV2 (ENSG00000166833) | chr11 | -1 | 20005730 | 5 | FALSE | CCTCTCTCAAAAGCCTGCCTG | TACTGCTGTCAGAAGCACCAC |
| OT6 | CGCCTACCTCACCAGTGGCAT | CTGGA | 0.113023477 |  | chr10 | -1 | 25408992 | 5 | FALSE | AAAGCACATAGCGGCGGAA | GAAACATGATGGGGGTGGGTA |
| OT7 | GCCATTCCACACCCATCGCAT | ATGGG | 0.109682882 |  | chr6 | -1 | 31333868 | 5 | FALSE | TGCATGCCAGCAGCTTCTTA | GCAACATGTTTTTACTCCCCACT |
| OT8 | AGCATACCACACCCACTGCAT | GAGGG | 0.108612805 |  | chr18 | 1 | 35109411 | 5 | FALSE | TGCTTGGATGGGTAACCTGG | AGTAGCCCCTCAGGACCAAA |
| OT9 | TGCGTACCACAGCCGCAGGAT | GAGAG | 0.092388485 |  | chr7 | 1 | 156183789 | 4 | FALSE | TGATGGAACTCAGCCCACG | GCTCATTGTCTTGCCATTTCGT |
| OT10 | TGCGTACATCATACGTCGCAT | CTGAA | 0.089420181 |  | chr8 | -1 | 37075079 | 4 | FALSE | CTTGAATGCACACAGGGTCA | CTCAGGAATCACAGGGAGATGG |

**Supplementary file 1e. Off-target sites of 17-Sg2 obtained from Cas-OFFinder (http://www.rgenome.net/cas-offinder) and NGS results.**

Red indicates mismatches of off-target sequence compared to 17-Sg2 sequence.

| Off-target ID | Sequence | PAM | Chromosome | Position | Strand | Mismatches | On-target | Forward primer | Reverse primer | Product length |
| --- | --- | --- | --- | --- | --- | --- | --- | --- | --- | --- |
| **N/A** | **TGCGTACCACACCCaTCGCAT** | **TGGAG** | **chr3** | **129247623** | **-1** | **0** | **TRUE** | **ATGAATGGCACAGAAGGCCC** | **GAGCAGGATGTAGTTGAGAGGC** | 231 |
| Off1 | TGAGTACCAGCCCCTGTCACAT | CTGAAT | chr1 | 203955786 | 1 | 4 | FALSE | AAAGGCATTCCTACCCCAAGA | CTGGGATGGGTGTGCAAATTC | 247 |
| Off2 | TGGGTACCACACCAGCTCTGAT | GGGGGT | chr1 | 198213041 | -1 | 4 | FALSE | GACAAAGCCAACACACTAAGGC | CCCTAAAGGCCAGACTACTGT | 223 |
| Off3 | GGCCTGTCCCACACCACTCGCAT | CAGGAT | chr1 | 111368178 | -1 | 4 | FALSE | ACAAAACAAAGAGGAGTGGAGAGT | TGCTTGCTGGATCCCAGTTT | 207 |
| Off4 | GGCCTCAGCCACACCCTTCGCAT | TAGAGT | chr2 | 7694251 | 1 | 4 | FALSE | TACCCATCCACGAGGATATACAGT | TGCAAGTTTCCTTTGGGGAAGT | 239 |
| Off5 | TGCCTGCCACACCCTGGAGGCAT | TCGGGT | chr2 | 121961972 | -1 | 4 | FALSE | AGGCATGCAGCAGTCTGG | AAGGCTGAACTTGGTCCTCG | 234 |
| Off6 | GGCGTGCCCCACCCTTCTGGCAT | ATGGAT | chr2 | 51489418 | 1 | 4 | FALSE | GAGTTCAAAGCACAGGAACTTC | TGTGTGACCAGACAGTTGGAAC | 240 |
| Off7 | TGAGCAACCACACACGTCGGAT | GTGAGT | chr6 | 11382305 | 1 | 4 | FALSE | GCTCATGTTTCCCTCTCCATGC | GAACAAGCTTTCTTACCTGTCCC | 249 |
| Off8 | TGCGTACCACAGCCGCAGGAT | GAGAGT | chr7 | 156391076 | 1 | 4 | FALSE | GGCAGCCAAGAAGCACCG | AGAATCCATCTCGTGGCTCG | 247 |
| Off9 | TGCCAGGGCCACTCCTGTCGCAT | CTGAGT | chr9 | 108166315 | 1 | 4 | FALSE | TGGTGTGGGGCCGTATCT | GTTAACTGTGTTTTACATCCTCTGG | 203 |
| Off10 | TGCTGTAACACACCAGCCTCAT | GTGAAT | chr10 | 75341442 | 1 | 4 | FALSE | GCACCCATAGGAAAAGTCTGTG | AATTCTCAGCCAACCCACAAGT | 222 |
| Off11 | TGCCTCTCCCACACCTGTGGCAT | CCGAGT | chr10 | 128566018 | 1 | 4 | FALSE | TGGCGGGAATATTTATCTACACCAT | CTTCCGTGGCAGAAAGCTACA | 216 |
| Off12 | TGCCTGCCACACCAGGGTGGCAT | TGGGGT | chr11 | 446447 | 1 | 4 | FALSE | GACCCCCAGCCTGAAGC | CTCTGGCTTGTTGGAGGGAG | 243 |
| Off13 | TGAGTACCACACCCACCACACT | GTGAAT | chr14 | 61376057 | -1 | 4 | FALSE | CTCCAGCCTGGGCAACAAGA | GCTACCCTGAGCAGACCTTC | 232 |
| Off14 | TGCTTGGCCACACCCGTCCCAT | CTGGGT | chr15 | 100384583 | -1 | 4 | FALSE | GGCAGAGACTCCACCAATCC | GAGCTTTGCTTCCTGGGACT | 246 |
| Off15 | TGCGTCACCACACCTGGCTAAT | TTGAAT | chr16 | 861503 | 1 | 3 | FALSE | GCTCACTGTAACCTCTGCCTC | TTATGAAGCGAGGCCGGG | 239 |
| Off16 | TGCGTCACCACACCTGGCCCAT | GTGAGT | chr16 | 10438533 | 1 | 4 | FALSE | CCCATGCTTCCCTCAAACTCC | GCCGATATTGCTAAGCTATAGT | 246 |
| Off17 | TGCGAACCAGACCCGTCACACTC | CGGGGT | chr17 | 60090564 | -1 | 4 | FALSE | GAGGTTGTGAAAACAAGCCTGA | CCCTGTGCCAGGGGTATGTA | 241 |
| Off18 | TGCGTGACACACCCATACCCAT | TAGGAT | chr19 | 51877543 | 1 | 4 | FALSE | ACCACAGCATTTGCACTTTGA | GTCAGTACCATTTTACATTCCCACC | 232 |
| Off19 | TGGGCACTCCACGCCCATCGCAT | GAGAGT | chr20 | 60038317 | 1 | 4 | FALSE | ATTGCAGACTCTTTTCAGATGGGA | GCAGAATCTGGCCTTCCGTC | 204 |
| Off20 | TAGGTACCACACCCAAGTCACCT | CTGGGT | chr20 | 4400103 | -1 | 4 | FALSE | CCATTTTTATACAGTTGGATTAGCAA | GTCCATTTGTCTGATCATTTTCTTAG | 231 |

**Supplementary file 1f. Off-target sites of 17-Sg2 obtained from Cas-OFFinder (http://www.rgenome.net/cas-offinder) and NGS results.**

| Sample ID | Off-target ID | Deletion_num | Insertion_Deletion_num | Insertion_num | NHEJ_num | Unmodified |
| --- | --- | --- | --- | --- | --- | --- |
| 293T-control | RHO-WT site | 441(0.0936%) | 2154(0.4574%) | 22832(4.8482%) | 25427(5.3992%) | 445513(94.6008%) |
| 293T-17-Sg2 | RHO-WT site | 750(0.0964%) | 3688(0.4743%) | 39864(5.1265%) | 44302(5.6972%) | 733310(94.3028%) |
| 293T-control | off1 | 2414(0.5363%) | 596(0.1324%) | 2370(0.5265%) | 5380(1.1952%) | 444761(98.8048%) |
| 293T-17-Sg2 | off1 | 2533(0.5124%) | 565(0.1143%) | 1735(0.3509%) | 4833(0.9776%) | 489553(99.0224%) |
| 293T-control | off2 | 812(0.2702%) | 664(0.2210%) | 363(0.1208%) | 1839(0.6120%) | 298657(99.3880%) |
| 293T-17-Sg2 | off2 | 690(0.2295%) | 618(0.2055%) | 295(0.0981%) | 1603(0.5331%) | 299103(99.4669%) |
| 293T-control | off3 | 162(0.0881%) | 156(0.0849%) | 8(0.0044%) | 326(0.1774%) | 183470(99.8226%) |
| 293T-17-Sg2 | off3 | 159(0.0903%) | 123(0.0699%) | 16(0.0091%) | 298(0.1693%) | 175698(99.8307%) |
| 293T-control | off4 | 378(0.1363%) | 1111(0.4007%) | 69(0.0249%) | 1558(0.5619%) | 275719(99.4381%) |
| 293T-17-Sg2 | off5 | 444(0.1450%) | 959(0.3133%) | 70(0.0229%) | 1473(0.4812%) | 304659(99.5188%) |
| 293T-control | off5 | 601(0.1010%) | 510(0.0857%) | 52(0.0087%) | 1163(0.1954%) | 593993(99.8046%) |
| 293T-17-Sg2 | off5 | 535(0.0896%) | 420(0.0703%) | 40(0.0067%) | 995(0.1666%) | 596146(99.8334%) |
| 293T-control | off6 | 2507(0.6303%) | 655(0.1647%) | 334(0.0840%) | 3496(0.8789%) | 394268(99.1211%) |
| 293T-17-Sg2 | off6 | 2385(0.6036%) | 616(0.1559%) | 255(0.0645%) | 3256(0.8240%) | 391898(99.1760%) |
| 293T-control | off7 | 1302(0.2401%) | 875(0.1614%) | 148(0.0273%) | 2325(0.4288%) | 539838(99.5712%) |
| 293T-17-Sg2 | off7 | 750(0.2310%) | 491(0.1512%) | 90(0.0277%) | 1331(0.4099%) | 323381(99.5901%) |
| 293T-control | off8 | 275(0.0511%) | 641(0.1191%) | 117(0.0217%) | 1033(0.1919%) | 537225(99.8081%) |
| 293T-17-Sg2 | off8 | 337(0.0502%) | 757(0.1127%) | 202(0.0301%) | 1296(0.1929%) | 670585(99.8071%) |
| 293T-control | off9 | 819(0.2224%) | 312(0.0847%) | 246(0.0668%) | 1377(0.3739%) | 366901(99.6261%) |
| 293T-17-Sg2 | off9 | 271(0.2241%) | 85(0.0703%) | 74(0.0612%) | 430(0.3556%) | 120494(99.6444%) |
| 293T-control | off10 | 20889(5.1968%) | 666(0.1657%) | 11941(2.9707%) | 33496(8.3332%) | 368463(91.6668%) |
| 293T-17-Sg2 | off10 | 23879(4.6622%) | 764(0.1492%) | 16917(3.3029%) | 41560(8.1143%) | 470620(91.8857%) |
| 293T-control | off11 | 719(0.2307%) | 258(0.0828%) | 46(0.0148%) | 1023(0.3283%) | 310578(99.6717%) |
| 293T-17-Sg2 | off11 | 670(0.2664%) | 174(0.0692%) | 36(0.0143%) | 880(0.3499%) | 250605(99.6501%) |
| 293T-control | off12 | 5363(0.9884%) | 657(0.1211%) | 249(0.0459%) | 6269(1.1553%) | 536352(98.8447%) |
| 293T-17-Sg2 | off12 | 5642(0.9764%) | 625(0.1082%) | 179(0.0310%) | 6446(1.1156%) | 571380(98.8844%) |
| 293T-control | off13 | 273376(74.9158%) | 43565(11.9385%) | 14268(3.9100%) | 331209(90.7643%) | 33702(9.2357%) |
| 293T-17-Sg2 | off13 | 300893(75.5530%) | 46414(11.6544%) | 14489(3.6381%) | 361796(90.8455%) | 36458(9.1545%) |
| 293T-control | off14 | 1522(0.2977%) | 591(0.1156%) | 213(0.0417%) | 2326(0.4549%) | 508970(99.5451%) |
| 293T-17-Sg2 | off14 | 1410(0.2728%) | 522(0.1010%) | 174(0.0337%) | 2106(0.4075%) | 514688(99.5925%) |
| 293T-control | off15 | 108(6.9813%) | 14(0.9050%) | 69(4.4602%) | 191(12.3465%) | 1356(87.6535%) |
| 293T-17-Sg2 | off15 | 111(6.7766%) | 13(0.7937%) | 67(4.0904%) | 191(11.6606%) | 1447(88.3394%) |
| 293T-control | off16 | 1870(0.4322%) | 553(0.1278%) | 618(0.1428%) | 3041(0.7028%) | 429666(99.2972%) |
| 293T-17-Sg2 | off16 | 2186(0.3894%) | 678(0.1208%) | 700(0.1247%) | 3564(0.6348%) | 557876(99.3652%) |
| 293T-control | off17 | 1021(0.1968%) | 386(0.0744%) | 2665(0.5136%) | 4072(0.7848%) | 514770(99.2152%) |
| 293T-17-Sg2 | off17 | 1112(0.2032%) | 356(0.0650%) | 3146(0.5748%) | 4614(0.8431%) | 542672(99.1569%) |
| 293T-control | off18 | 4269(0.8299%) | 1741(0.3385%) | 2526(0.4911%) | 8536(1.6594%) | 505853(98.3406%) |
| 293T-17-Sg2 | off18 | 3570(0.7832%) | 1360(0.2984%) | 2405(0.5276%) | 7335(1.6092%) | 448484(98.3908%) |
| 293T-control | off19 | 1988(0.4913%) | 194(0.0479%) | 123(0.0304%) | 2305(0.5696%) | 402372(99.4304%) |
| 293T-17-Sg2 | off19 | 1164(0.4907%) | 133(0.0561%) | 66(0.0278%) | 1363(0.5746%) | 235843(99.4254%) |
| 293T-control | off20 | 1086(0.2307%) | 719(0.1527%) | 488(0.1037%) | 2293(0.4871%) | 468445(99.5129%) |
| 293T-17-Sg2 | off20 | 1033(0.2452%) | 589(0.1398%) | 504(0.1196%) | 2126(0.5046%) | 419231(99.4954%) |

| **Supplementary file 1g. The number of off-target sites of 17-Sg2 obtained from Cas-OFFinder (<http://www.rgenome.net/cas-offinder>).** | | |
| --- | --- | --- |
| mismatches_num | candidate off-target sites_num | off-target sites validated by WGS_num |
| 1 | 0 | 0 |
| 2 | 0 | 0 |
| 3 | 6 | 0 |
| 4 | 42 | 0 |
| 5 | 351 | 9 |

**Supplementary file 1h. Whole-genome Sequencing results of off-target activity for SaCas9/17Sg-2.**

| #CHROM | POS | ID | REF | ALT | QUAL | Func | Gene | ExonicFunc | AAchange | 293T-17Sg-2. variant_Info(HomoHete;Depth;Frequency) | 293T.variant2_Info(HomoHete;AltDepth;Frequency) | MutType | MutLength | PosInfo | DNA | crRNA | Mismathes | BugleInfo | Distance |
| --- | --- | --- | --- | --- | --- | --- | --- | --- | --- | --- | --- | --- | --- | --- | --- | --- | --- | --- | --- |
| chr2 | 41773507 | rs70959463 | CGTGTGTGT | C,CGTGTGTGTGTGT | 49.92 | intergenic | SLC8A1;LINC01913 | . | . | Hete;9;0.474 | Hete;8,8;0.364,0.364 | InDel | -8,4 | 41773482;- | caCGTACACACACatATCGCAgATGTGA | TGCGTAC-CACACCCATCGCATNNGYGA | 5 | DNA;1 | 25 |
| chr2 | 176462528 | . | AT | A | 22.13 | intergenic | ATP5MC3;LNPK | . | . | Hete;9;0.600 | Homo;14;0.667 | InDel | -1 | 176462417;- | TGCacACCTACAgCCATCtaATCTGTGA | TGCGTACC-ACACCCATCGCATNNGYGA | 5 | DNA;1 | 111 |
| chr6 | 33110133 | rs9296085 | A | G | 119.21 | upstream | HCG24 | . | . | Homo;24;1.000 | Homo;26;1.000 | SNP | 0 | 33110189;- | TGGgGcACCACACCCAgCcCgTTAGTGA | TG-CGTACCACACCCATCGCATNNGYGA | 5 | DNA;1 | -56 |
| chr6 | 33110141 | rs35402346 | GA | G | 110.18 | upstream | HCG24 | . | . | Homo;21;1.000 | Homo;25;1.000 | InDel | -1 | 33110189;- | TGGgGcACCACACCCAgCcCgTTAGTGA | TG-CGTACCACACCCATCGCATNNGYGA | 5 | DNA;1 | -48 |
| chr8 | 37989344 | . | CTT | CT,C | 83.98 | intronic | ASH2L | . | . | Hete;7,7;0.444,0.444 | Hete;16,7;0.593,0.296 | InDel | -1,-2 | 37989401;- | TGCGTACCACtgCacTCCAGCcTGGGCGA | TGCGTACCACACCCATC--GCATNNGYGA | 5 | DNA;2 | -57 |
| chr9 | 86279838 | . | CAAAA | C,CA,CAA | 86.62 | intronic | UBQLN1 | . | . | Hete;7,7;0.467,0.467 | Hete;3,5;0.333,0.417 | InDel | -4,-3 | 86279787;+ | TGCGCccCCACACtCATaGCcTAGGCGA | TGCG-TACCACACCCATCGCATNNGYGA | 5 | DNA;1 | 51 |
| chr12 | 95426924 | rs200022502 | CAA | CA,C | 78.61 | intronic | NR2C1 | . | . | Homo;18;0.900 | Hete;6,8;0.375,0.500 | InDel | -1,-2 | 95426846;+ | gGgGTAgCACACCATgATtGCATCTGTGA | TGCGTACCACACC--CATCGCATNNGYGA | 5 | DNA;2 | 78 |
| chr13 | 46880587 | . | CTTTT | CTTT,C,CT,CTTTTT | 62.33 | intergenic | LINC00563;RUBCNL | . | . | Hete;6,9;0.333,0.476 | Hete;16,5;0.593,0.222 | InDel | -1,-4 | 46880535;+ | TGaGCcACCACACCCAgCctATATGTGA | TGCG-TACCACACCCATCGCATNNGYGA | 5 | DNA;1 | 52 |
| chr15 | 43834499 | rs35106975 | CG | C | 64.16 | intronic | PPIP5K1 | . | . | Homo;8;0.889 | Hete;7;0.636 | InDel | -1 | 43834526;- | atCGTACCACAgCacTCTAGCATGGGTGA | TGCGTACCACACCCATC--GCATNNGYGA | 5 | DNA;2 | -27 |

| **Supplementary file 1i. List of primers.** | |
| --- | --- |
| Primers | sequence (5`-3`) |
| **Oligos for molecular cloning and site-directed mutagenesis** | |
| FUGW-RHO F | CTGCAGGTCGACTCTAGAGGATCCATGAATGGCACAGAA |
| FUGW-RHO R | CGATAAGCTTGATATCGAATTTTAGGCCGGGGCCAC |
| RHO-pEGFPN1 F | GATCTCGAGCTCAAGCTTCGATGAATGGCACAGAAGGCC |
| RHO-pEGFPN1 R | CTTGCTCACCATGGTGGCGATGGCCGGGGCCACCTGGCT |
| c.46del F | CTACGTGCCCTTCTCCAATCGATGGGTGTGGTA |
| c.46del R | CCACACCCATCGATTGGAGAAGGGCACGTAGAAGTT |
| c.45_46del F | TAACTTCTACGTGCCCTTCTCCAACGATGGGTGTGGTAC |
| c.45_46del R | GTACCACACCCATCGTTGGAGAAGGGCACGTAGAA |
| c.44_46del F | TACGTGCCCTTCTCCACGATGGGTGTGGTACGCAGC |
| c.44_46del R | TACCACACCCATCGTGGAGAAGGGCACGTAGAAGTTAGG |
| c.43_46del F | TTCTACGTGCCCTTCTCCCGATGGGTGTGGTACGCA |
| c.43_46del R | TACCACACCCATCGGGAGAAGGGCACGTAGAAGTTAGG |
| c.46_55del F | TTCTACGTGCCCTTCTCCAATTGGTACGCAGCCCCTTC |
| c.46_55del R | AGGGGCTGCGTACCAATTGGAGAAGGGCACGTAGAAGTTAG |
| c.46_47ins49bp F | CAGTGAGCGAGCGAGCGCGCAGAGAGGGAGTGGCCAACGATGGGTGTGGTACGCAGC |
| c.46_47ins49bp R | CTCTGCGCGCTCGCTCGCTCACTGAGGCCGGGCGACCATTGGAGAAGGGCACGTAGAAG |
| **Oligos for humanized mice genotyping** | |
| WT-F | GGCAGCAGTGGGATTAGCGTTAGTA |
| WT-R | TGTGTAGAGGGTGGTGGTGAATCCT |
| Mut-R | ACGATCAGCAGAAACATGTAGGCGG |
| **Oligos for T7E1 cleavage analysis** | |
| RHO17-CDS F | GCAGTGCACCCGTACCTTT |
| RHO17-CDS R | TGCAGAGAGGTGTAGAGGGTG |
| RHO17-F | ATTATGAACACCCCCAATCTCC |
| RHO17-R | GGCTTTGGATAACATTGACAGGA |
| WT-F | GGCAGCAGTGGGATTAGCGTTAGTA |
| Rho-mR | TCTGAACCCATGTTTCTTGC |
| **Oligos for Hi-Tom sequencing** | |
| RHO17-Hi-Tom F | GGAGTGAGTACGGTGTGCATGGCACAGAAGGCCCTAAC |
| RHO17-Hi-Tom R | GAGTTGGATGCTGGATGGAGCGTGAGGAAGTTGATGGG |
| **Oligos for quantitative PCR** | |
| SaCas9 F | TACGAAGTGAATAGCAAGT |
| SaCas9 R | TGTAGAAGGAGGCGATAA |
| SgRNA17 F | TACCACACCCATCGCATGT |
| SgRNA R | TTGACGAGATAAACACGGCATTT |
| Human GAPDH F | CTCTGGTAAAGTGGATATTGT |
| Human GAPDH R | GGTGGAATCATATTGGAACA |
| Mouse Gapdh F | AGTGGCAAAGTGGAGATT |
| Mouse Gapdh R | GTGGAGTCATACTGGAACA |
| Macaca GAPDH F | TTGCCCTCAACGACCACTTT |
| Macaca GAPDH R | GAGGGGAGATTCAGTGTGGC |
| hRHO F | GTGCCCTTCTCCAATGCGA |
| hRHO R | TGAGGAAGTTGATGGGGAAGC |
| mRho F | TGAGGTCAACAACGAATC |
| mRho R | CCATAGCAGAAGAAGATGA |
