# Supplementary figures and images for: Allele-specific gene-editing approach for vision loss restoration in RHO-associated retinitis pigmentosa
Source: eLife. 2023 Jun 5;12:e84065. doi: 10.7554/eLife.84065 (PMC10279453; doi:10.7554/eLife.84065)

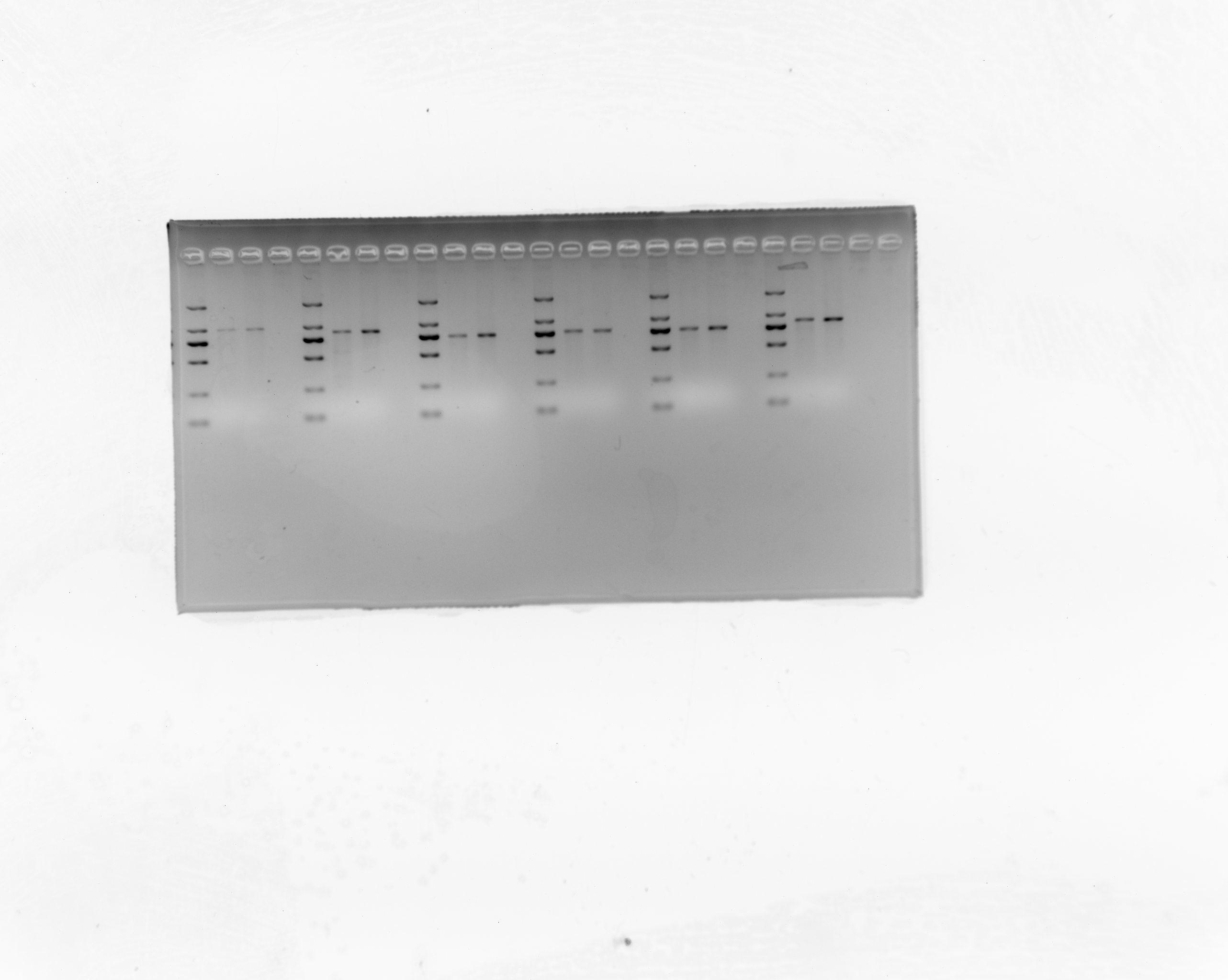

Supplement: Source data 1. [file elife-84065-data1.zip › Figure-Source Data 1/Figure 10-figure supplement 1-Source Data/Figure 10-figure supplement 1-Source Data2.tif]

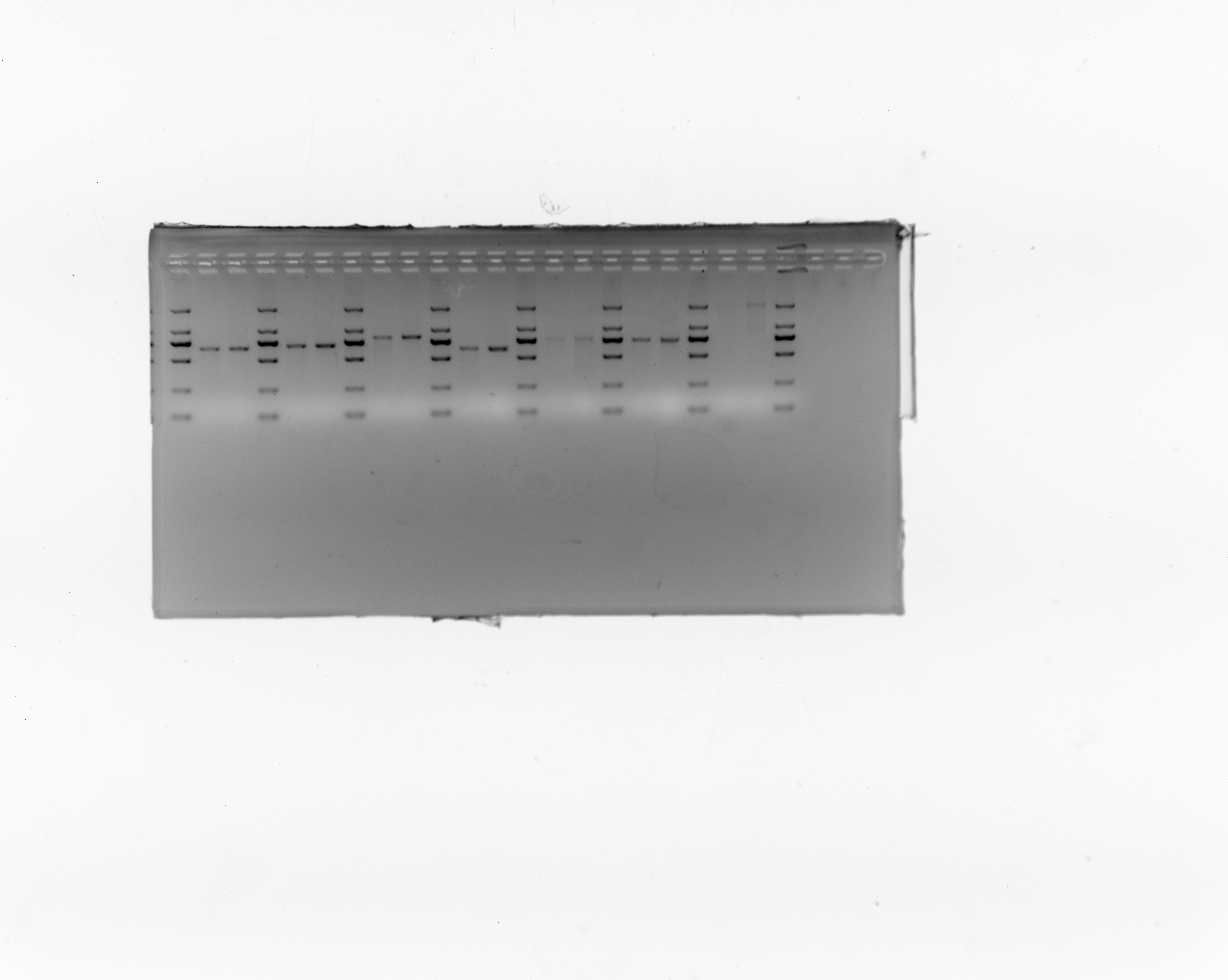

Supplement: Source data 1. [file elife-84065-data1.zip › Figure-Source Data 1/Figure 10-figure supplement 1-Source Data/Figure 10-figure supplement 1-Source Data3.tif]

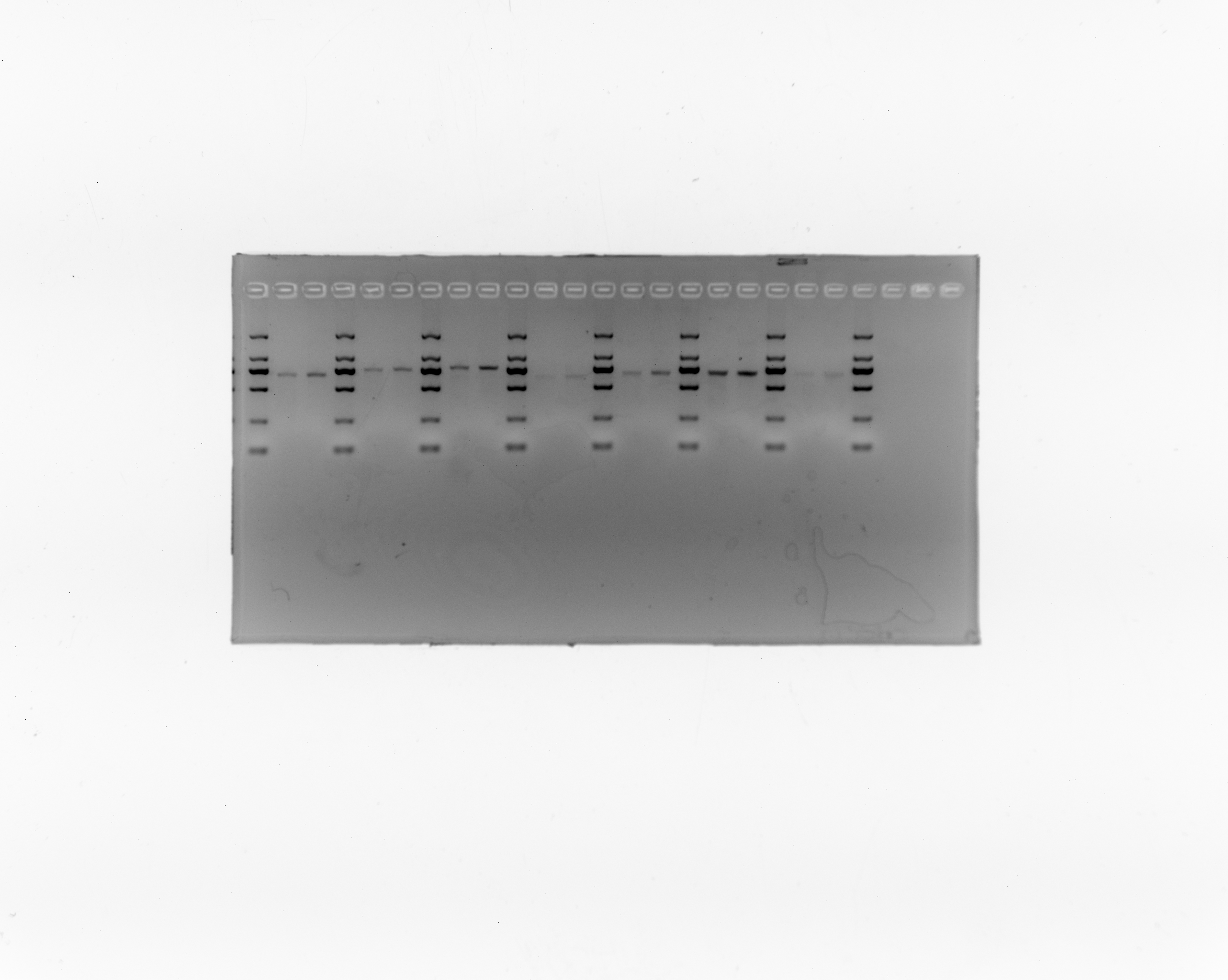

Supplement: Source data 1. [file elife-84065-data1.zip › Figure-Source Data 1/Figure 10-figure supplement 1-Source Data/Figure 9-figure supplement 1-Source Data1.tif]

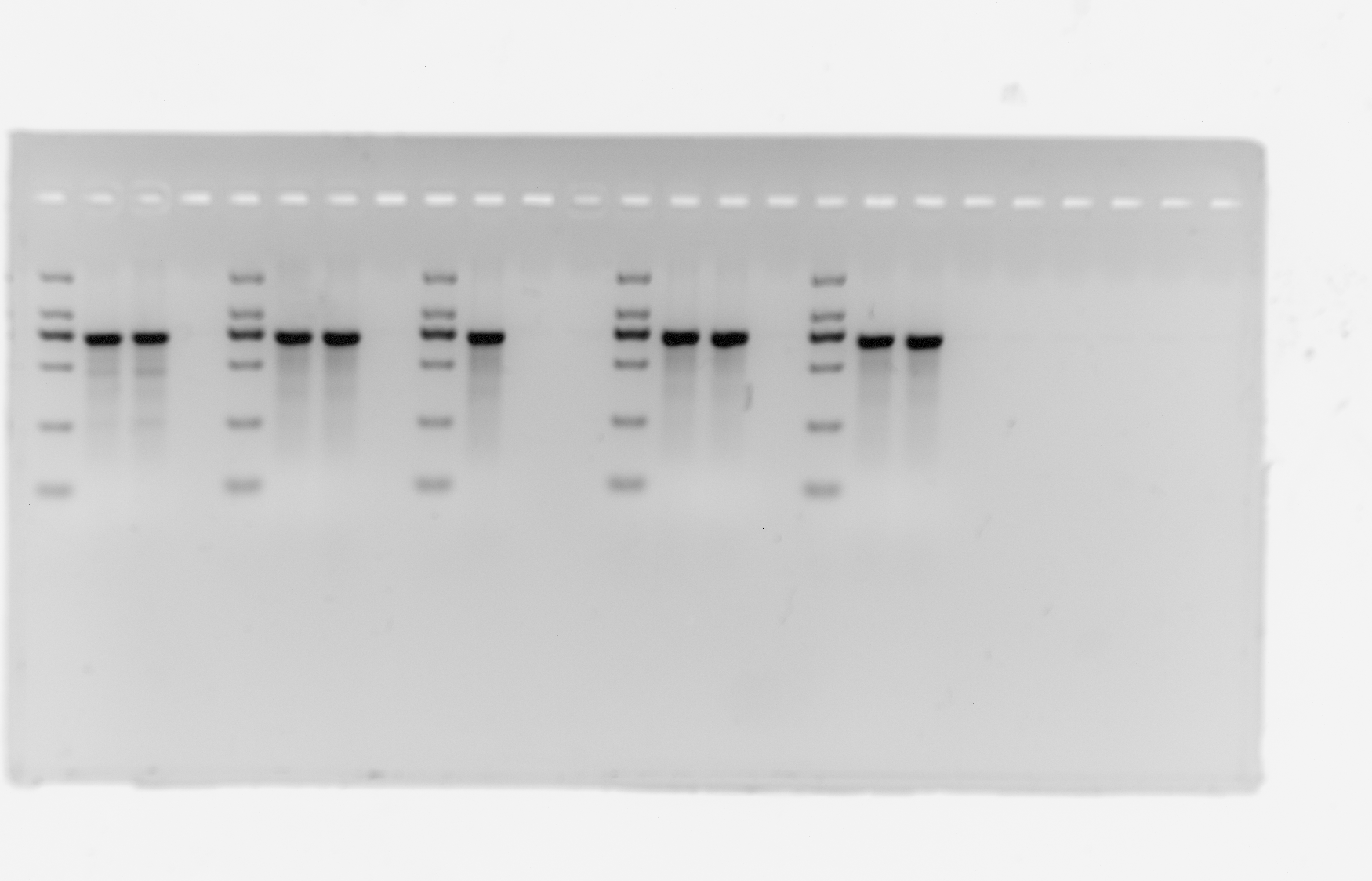

Supplement: Source data 1. [file elife-84065-data1.zip › Figure-Source Data 1/Figure 5-figure supplement 1-Source Data/Figure 5-figure supplement 1-Source Data1.tif]

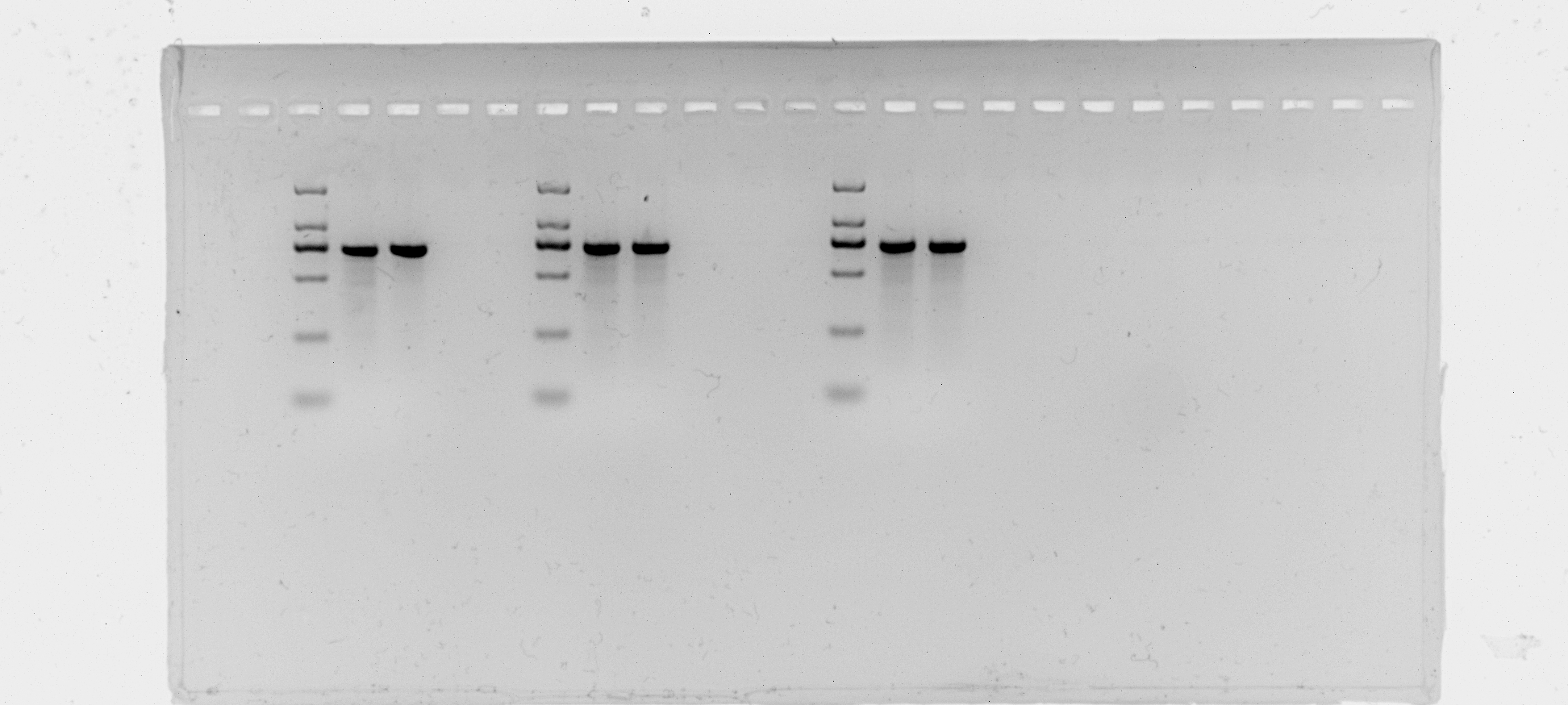

Supplement: Source data 1. [file elife-84065-data1.zip › Figure-Source Data 1/Figure 5-figure supplement 1-Source Data/Figure 5-figure supplement 1-Source Data2.tif]

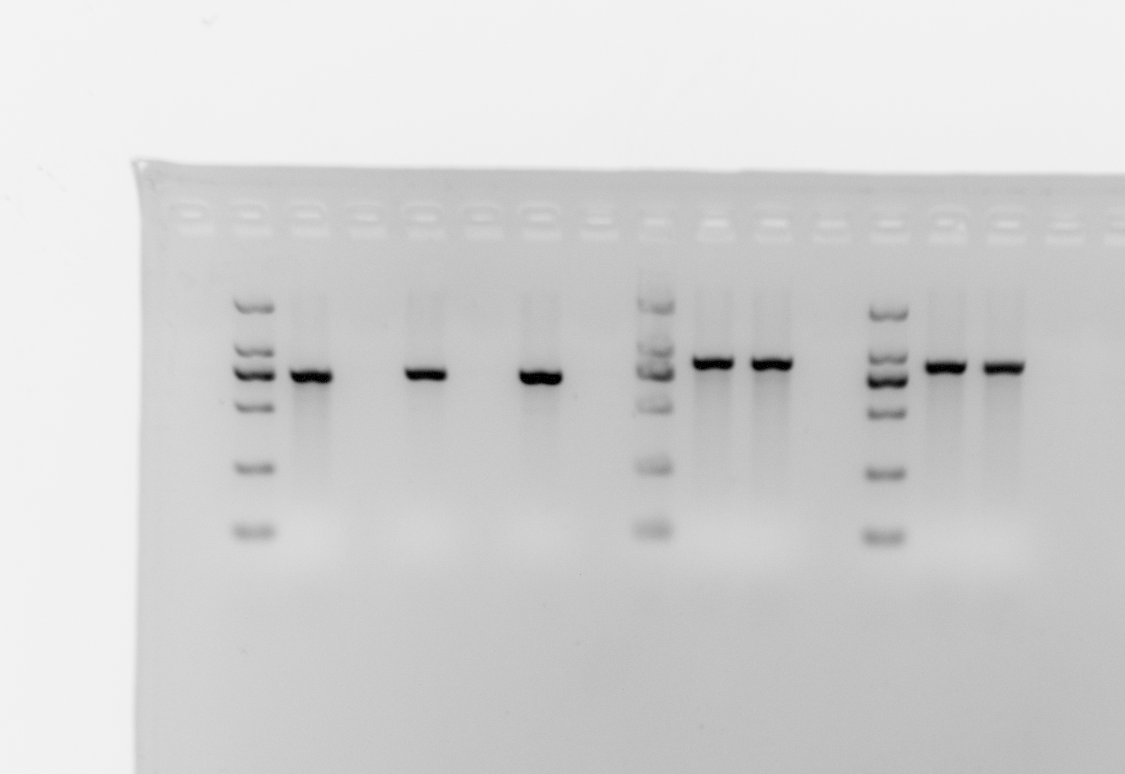

Supplement: Source data 1. [file elife-84065-data1.zip › Figure-Source Data 1/Figure 5-figure supplement 1-Source Data/Figure 5-figure supplement 1-Source Data3.tif]

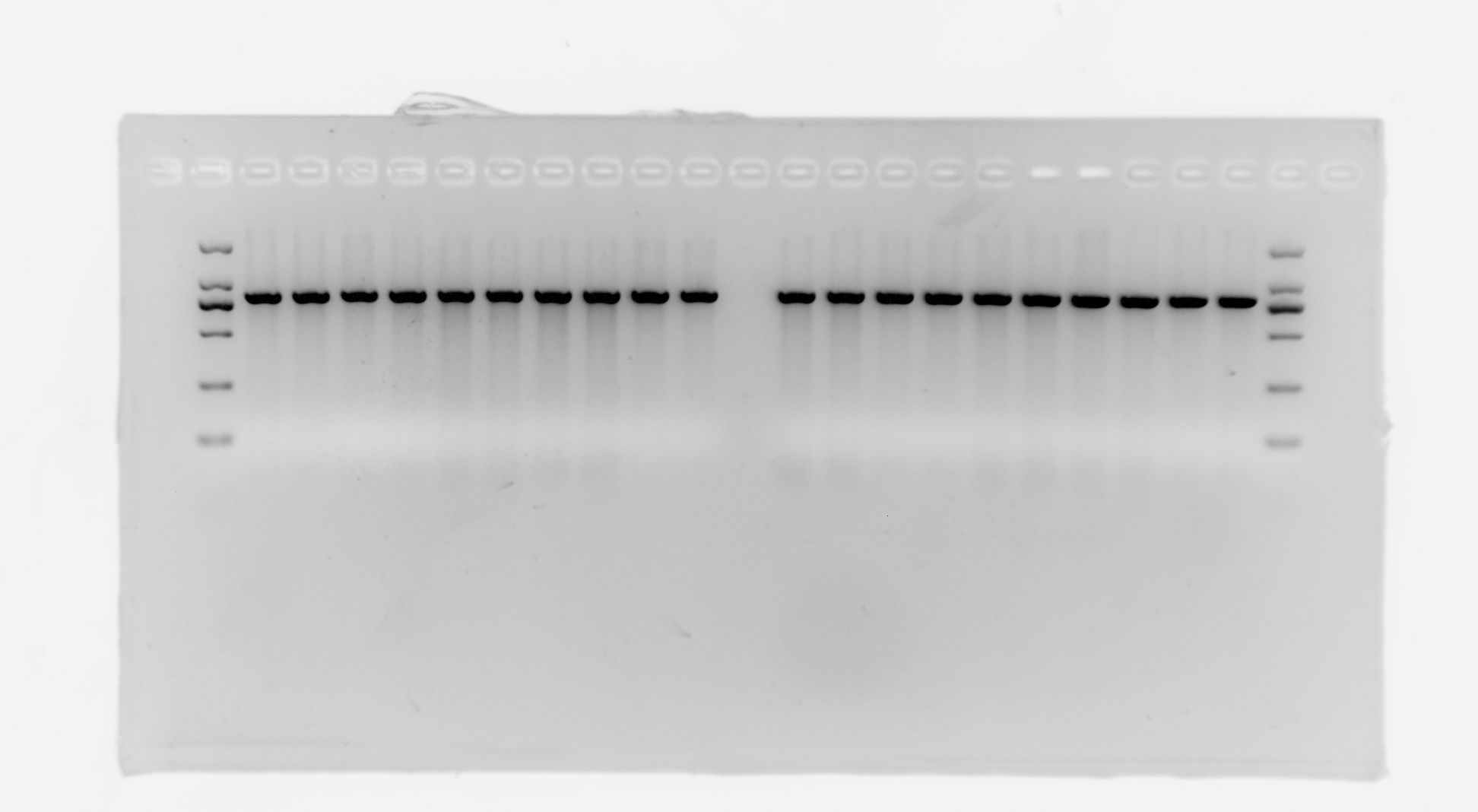

Supplement: Source data 1. [file elife-84065-data1.zip › Figure-Source Data 1/Figure 5-figure supplement 1-Source Data/Figure 5-figure supplement 1-Source Data4.tif]

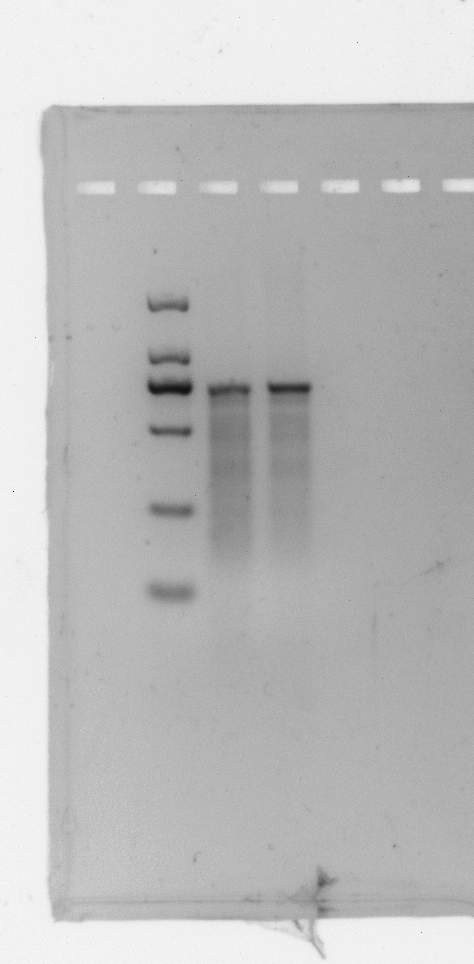

Supplement: Source data 1. [file elife-84065-data1.zip › Figure-Source Data 1/Figure 5-figure supplement 1-Source Data/Figure 5-figure supplement 1-Source Data5.tif]

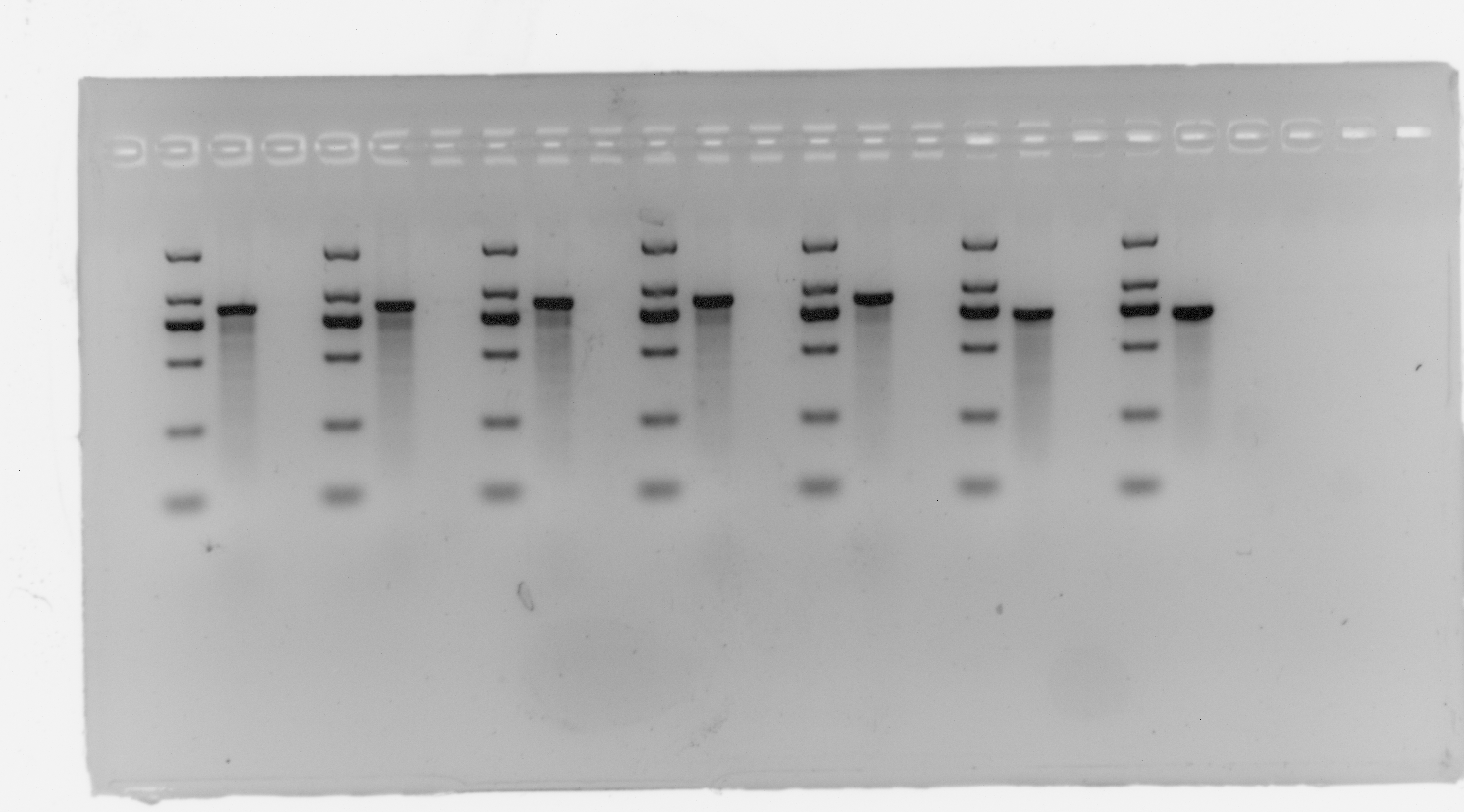

Supplement: Source data 1. [file elife-84065-data1.zip › Figure-Source Data 1/Figure 5-figure supplement 1-Source Data/Figure 5-figure supplement 1-Source Data6.tif]

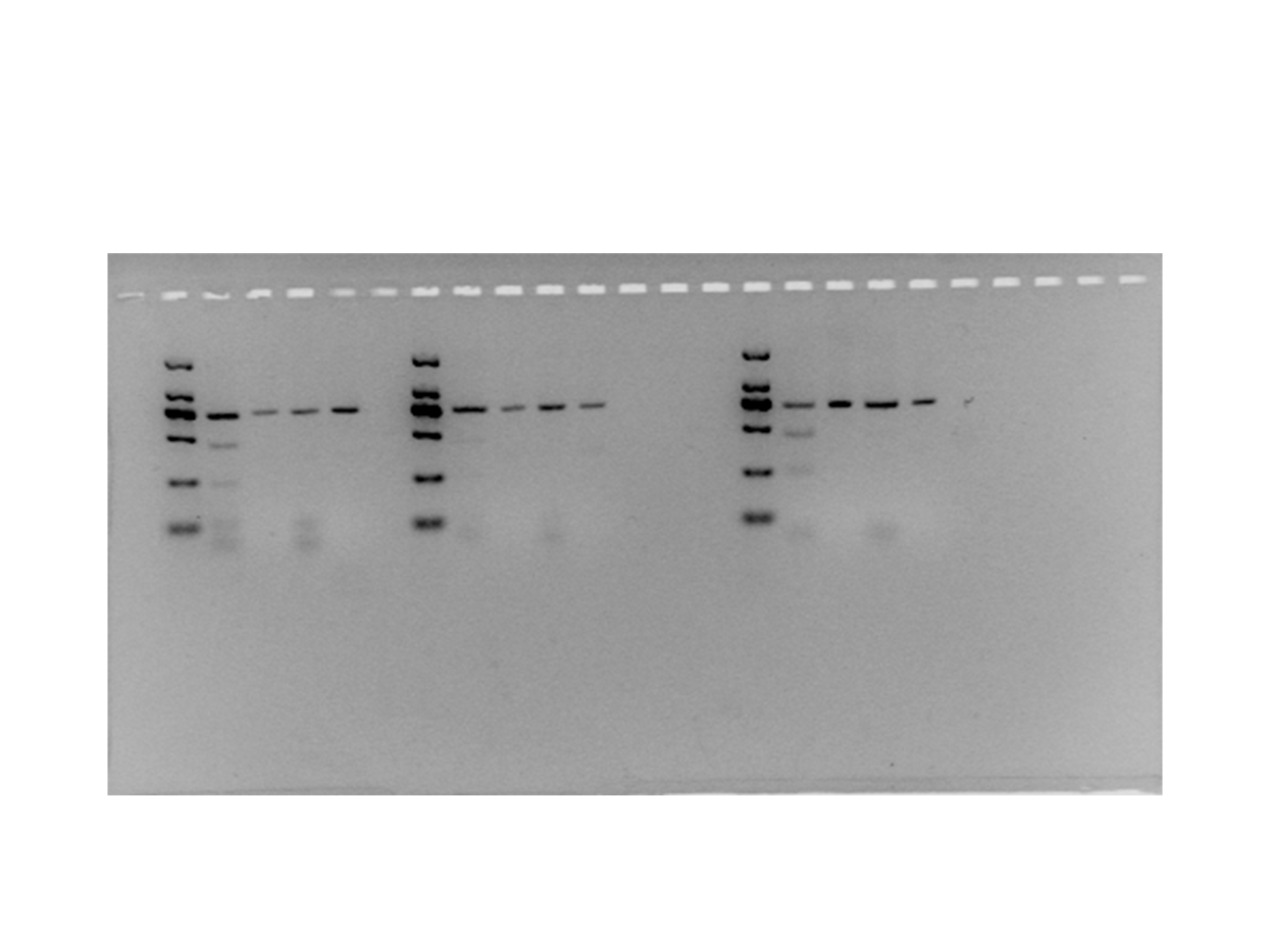

Supplement: Source data 1. [file elife-84065-data1.zip › Figure-Source Data 1/Figure1-Source Data/Figure 1-Source Data1.tif]

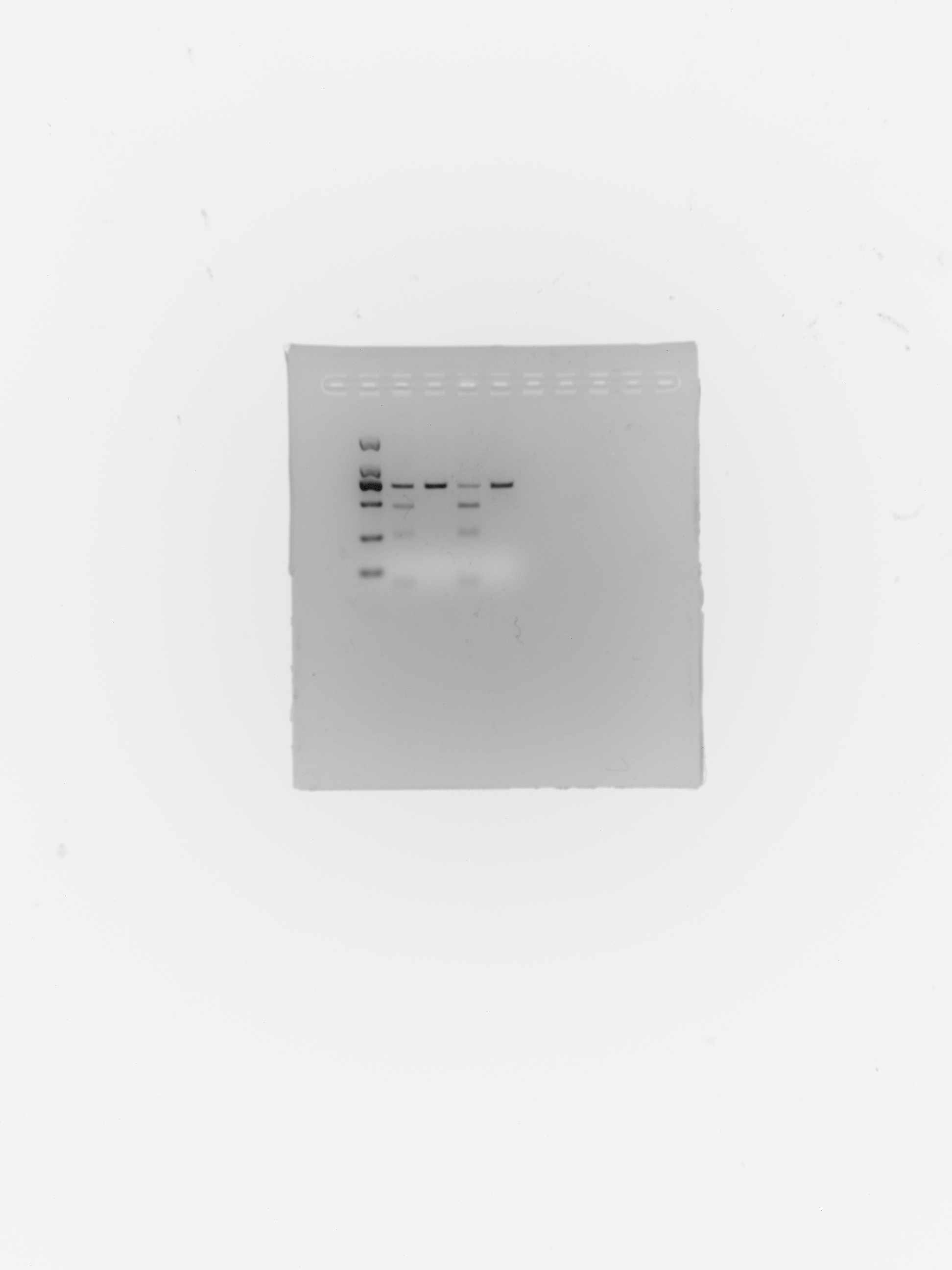

Supplement: Source data 1. [file elife-84065-data1.zip › Figure-Source Data 1/Figure1-Source Data/Figure 1-Source Data2.tif]

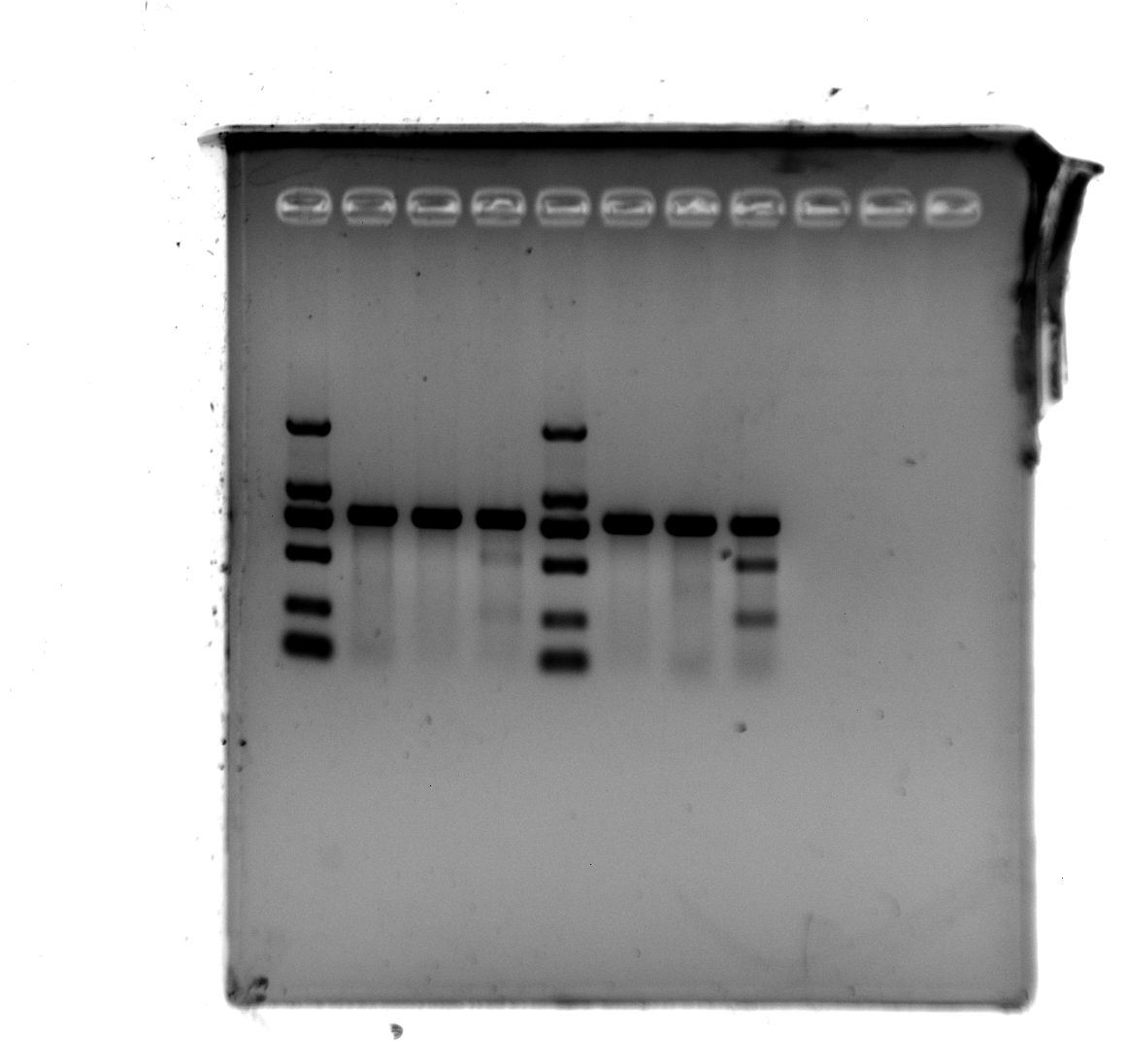

Supplement: Source data 1. [file elife-84065-data1.zip › Figure-Source Data 1/Figure2-Source Data/Figure 2-Source Data1.tif]

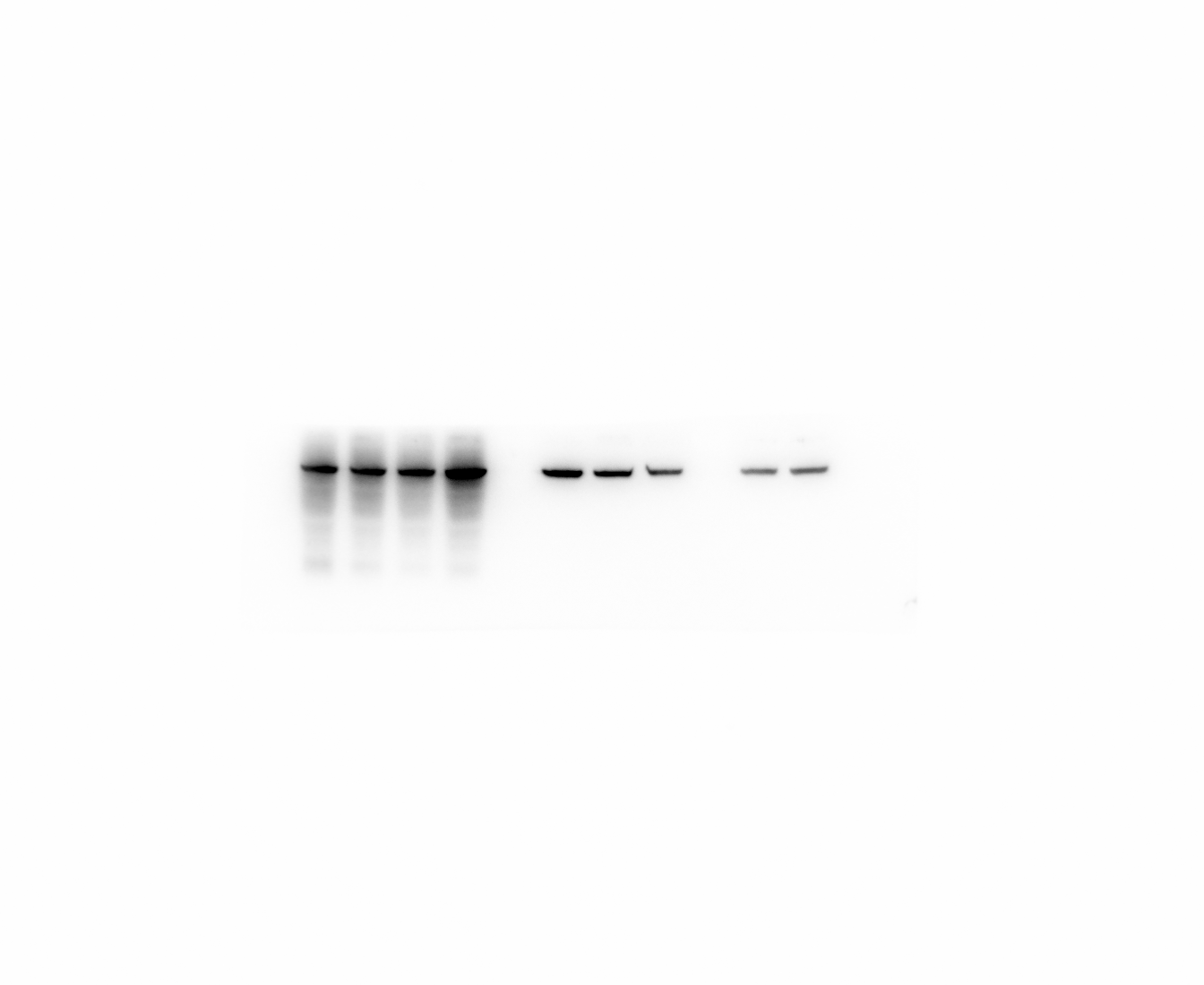

Supplement: Source data 1. [file elife-84065-data1.zip › Figure-Source Data 1/Figure2-Source Data/Figure 2-Source Data2.tif]

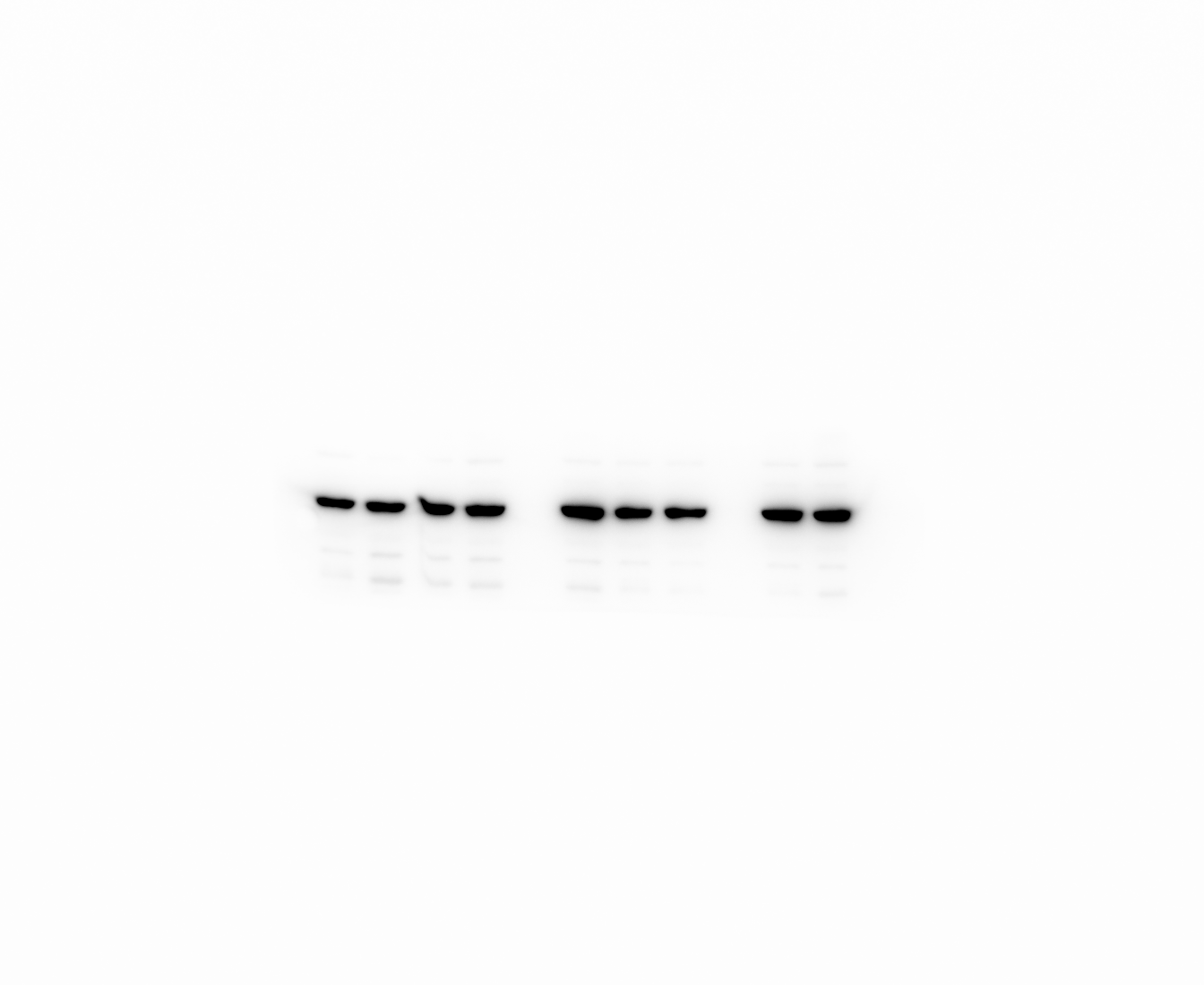

Supplement: Source data 1. [file elife-84065-data1.zip › Figure-Source Data 1/Figure2-Source Data/Figure 2-Source Data3.tif]

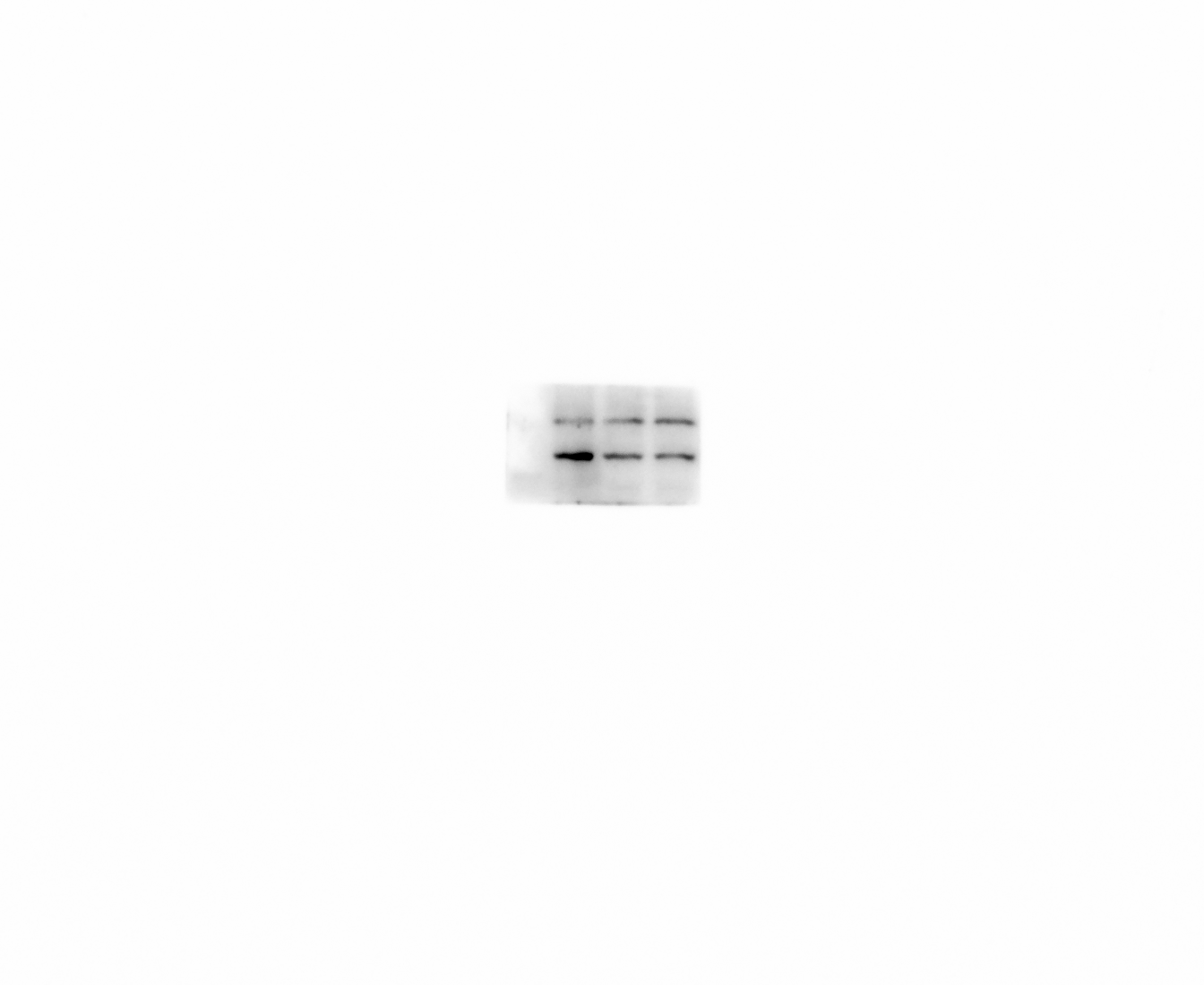

Supplement: Source data 1. [file elife-84065-data1.zip › Figure-Source Data 1/Figure2-Source Data/Figure 2-Source Data4.tif]

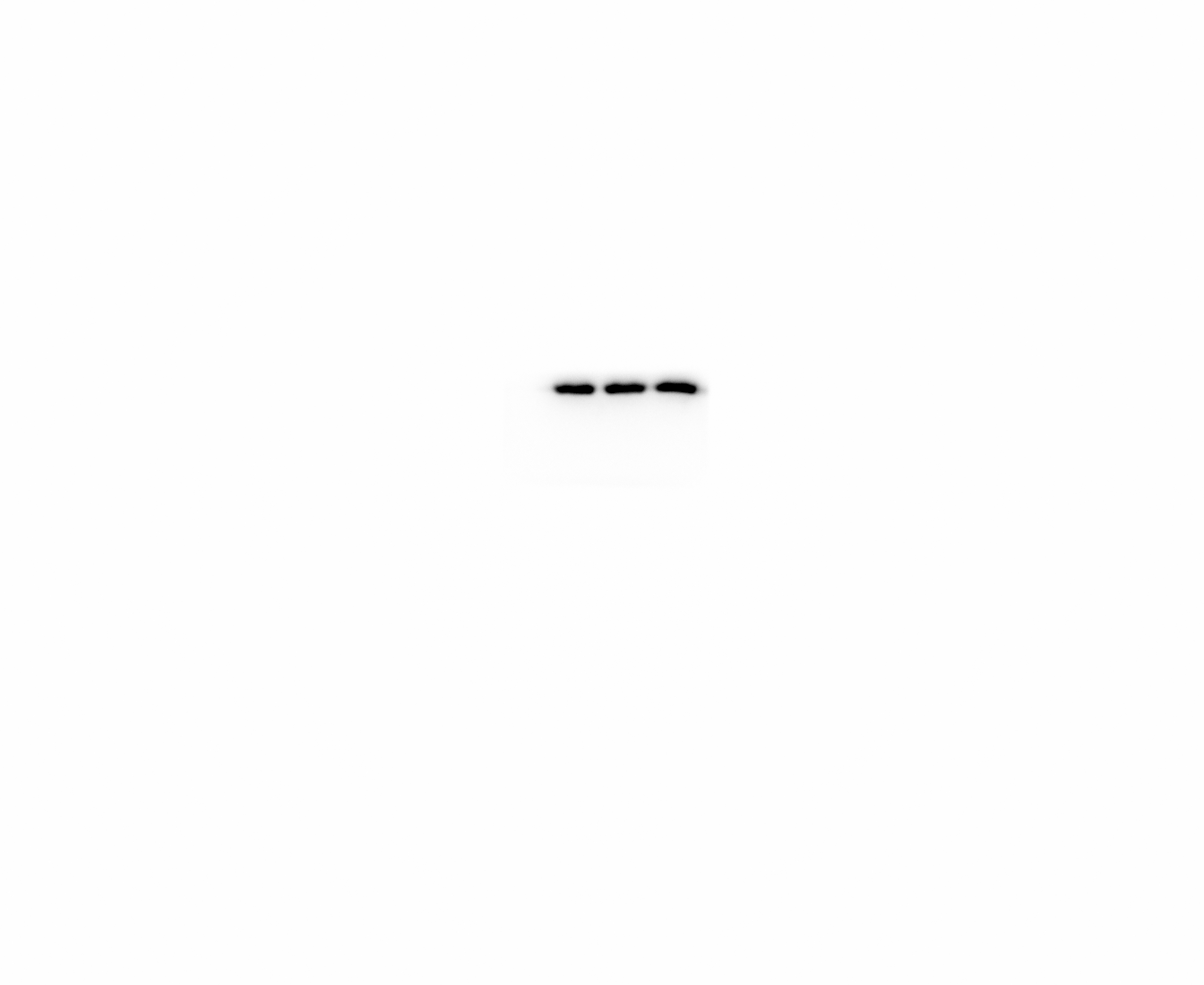

Supplement: Source data 1. [file elife-84065-data1.zip › Figure-Source Data 1/Figure2-Source Data/Figure 2-Source Data5.tif]

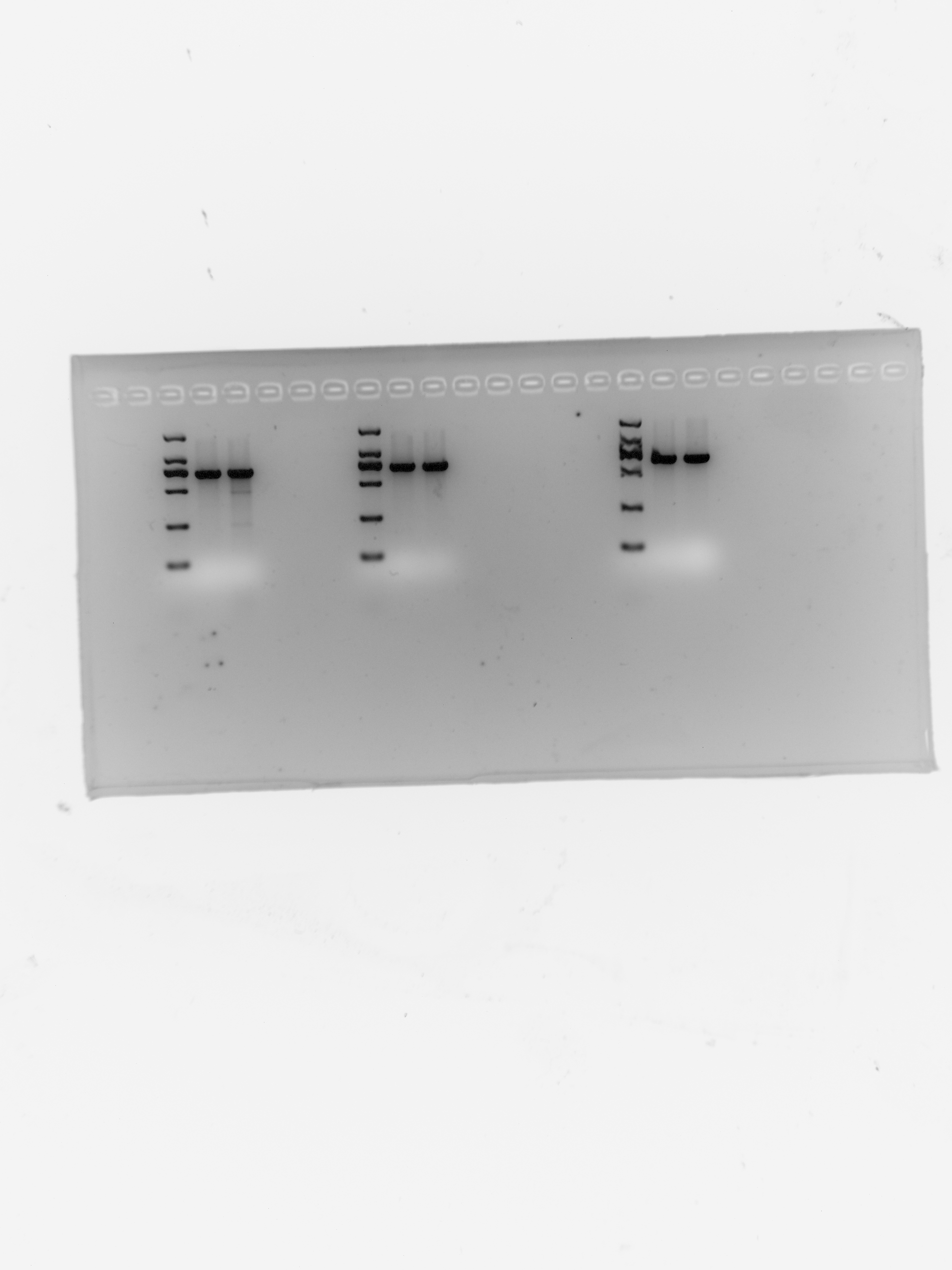

Supplement: Source data 1. [file elife-84065-data1.zip › Figure-Source Data 1/Figure3-Source Data/Figure 3-Source Data1.tif]

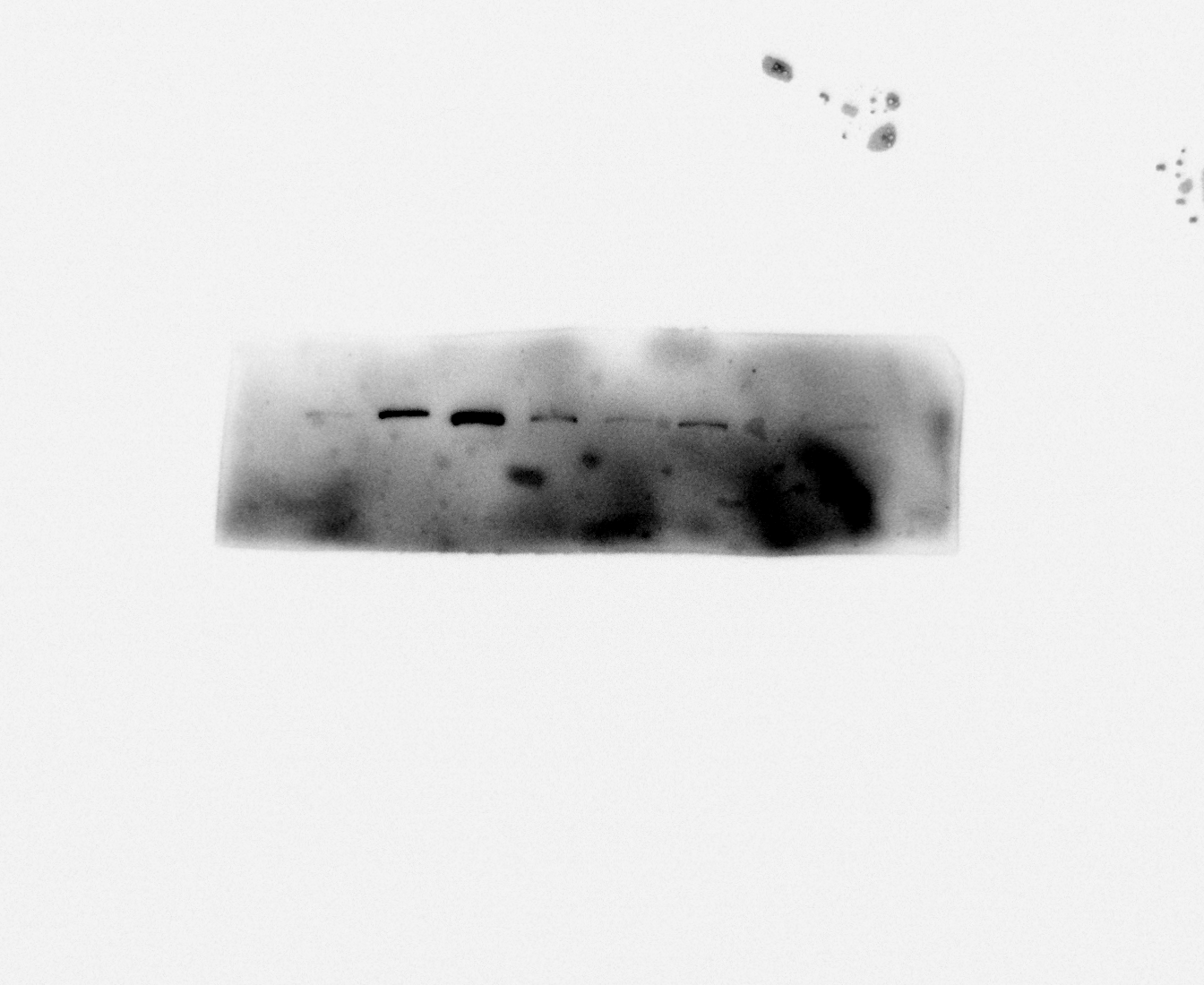

Supplement: Source data 1. [file elife-84065-data1.zip › Figure-Source Data 1/Figure4-Source Data/Figure4-Source Data1.tif]

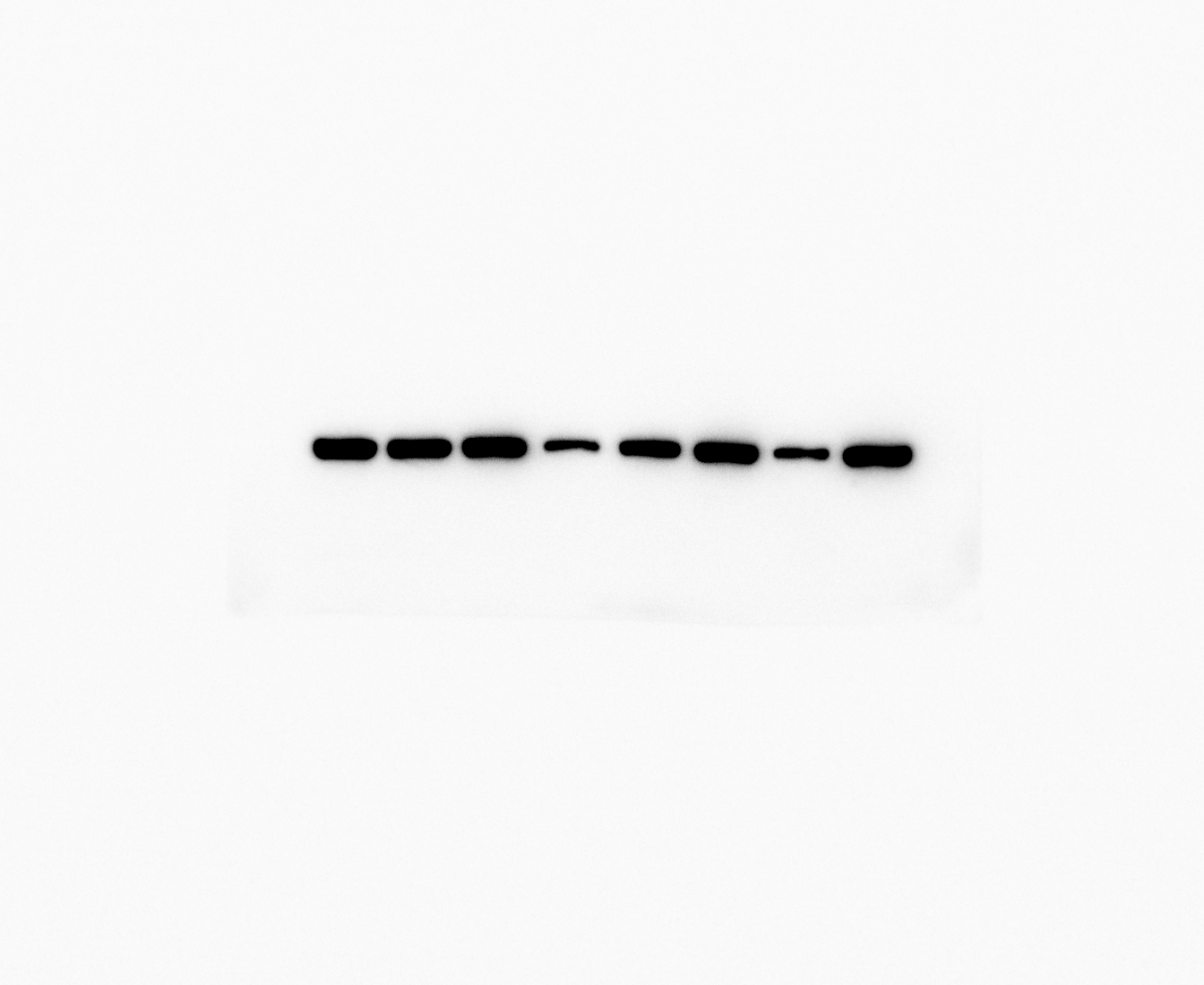

Supplement: Source data 1. [file elife-84065-data1.zip › Figure-Source Data 1/Figure4-Source Data/Figure4-Source Data2.tif]

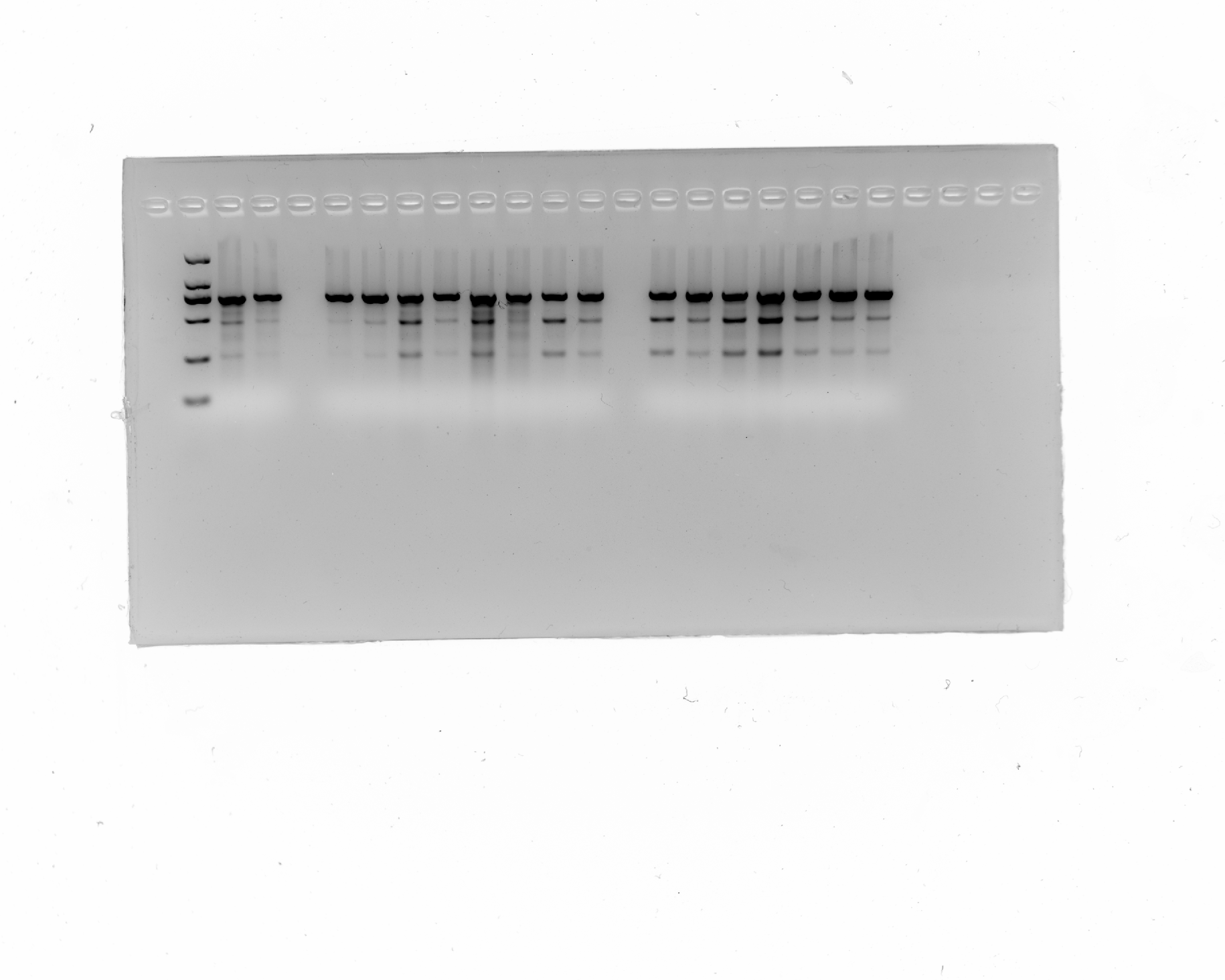

Supplement: Source data 1. [file elife-84065-data1.zip › Figure-Source Data 1/Figure5-Source Data/Figure 5-Source Data1.tif]

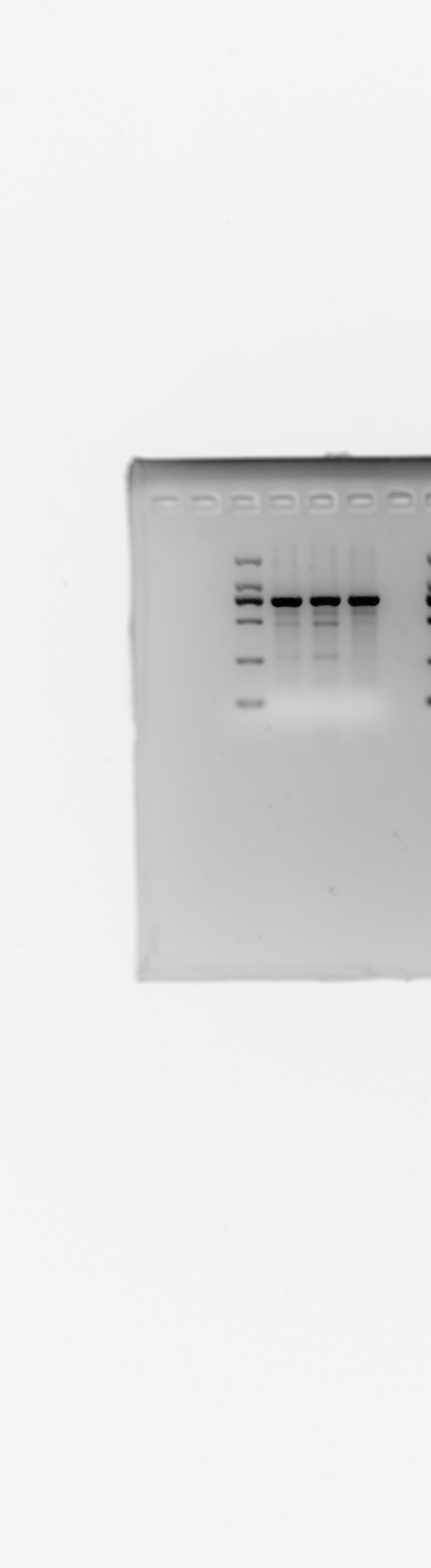

Supplement: Source data 1. [file elife-84065-data1.zip › Figure-Source Data 1/Figure7-Source Data/Figure 7-Source Data1.tif]

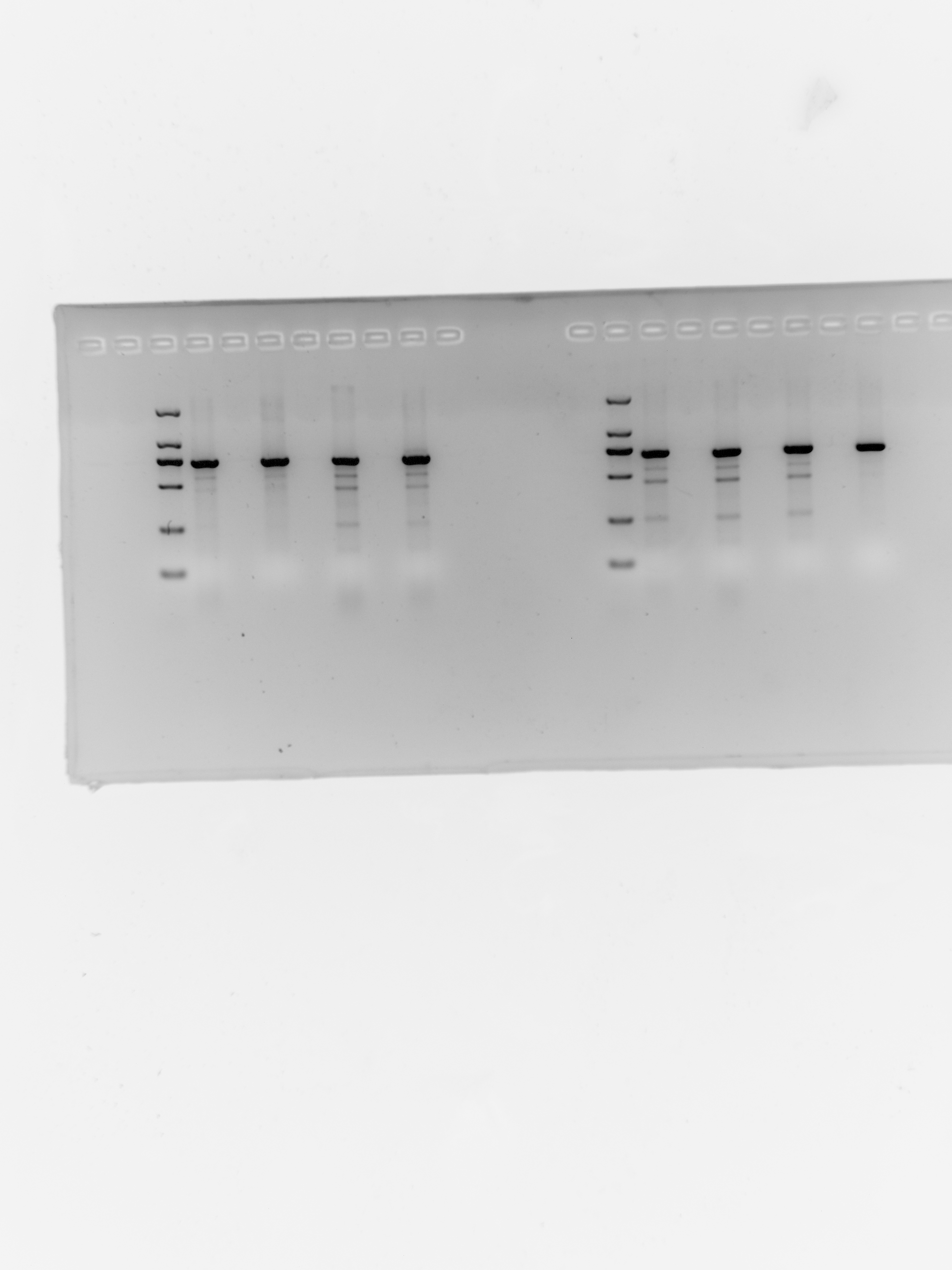

Supplement: Source data 1. [file elife-84065-data1.zip › Figure-Source Data 1/Figure7-Source Data/Figure 7-Source Data2.tif]

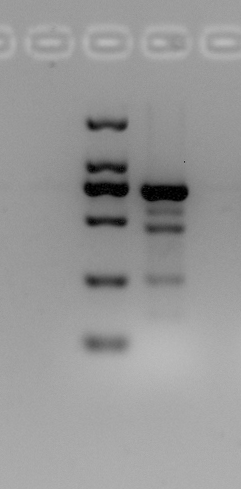

Supplement: Source data 1. [file elife-84065-data1.zip › Figure-Source Data 1/Figure7-Source Data/Figure 7-Source Data3.tif]

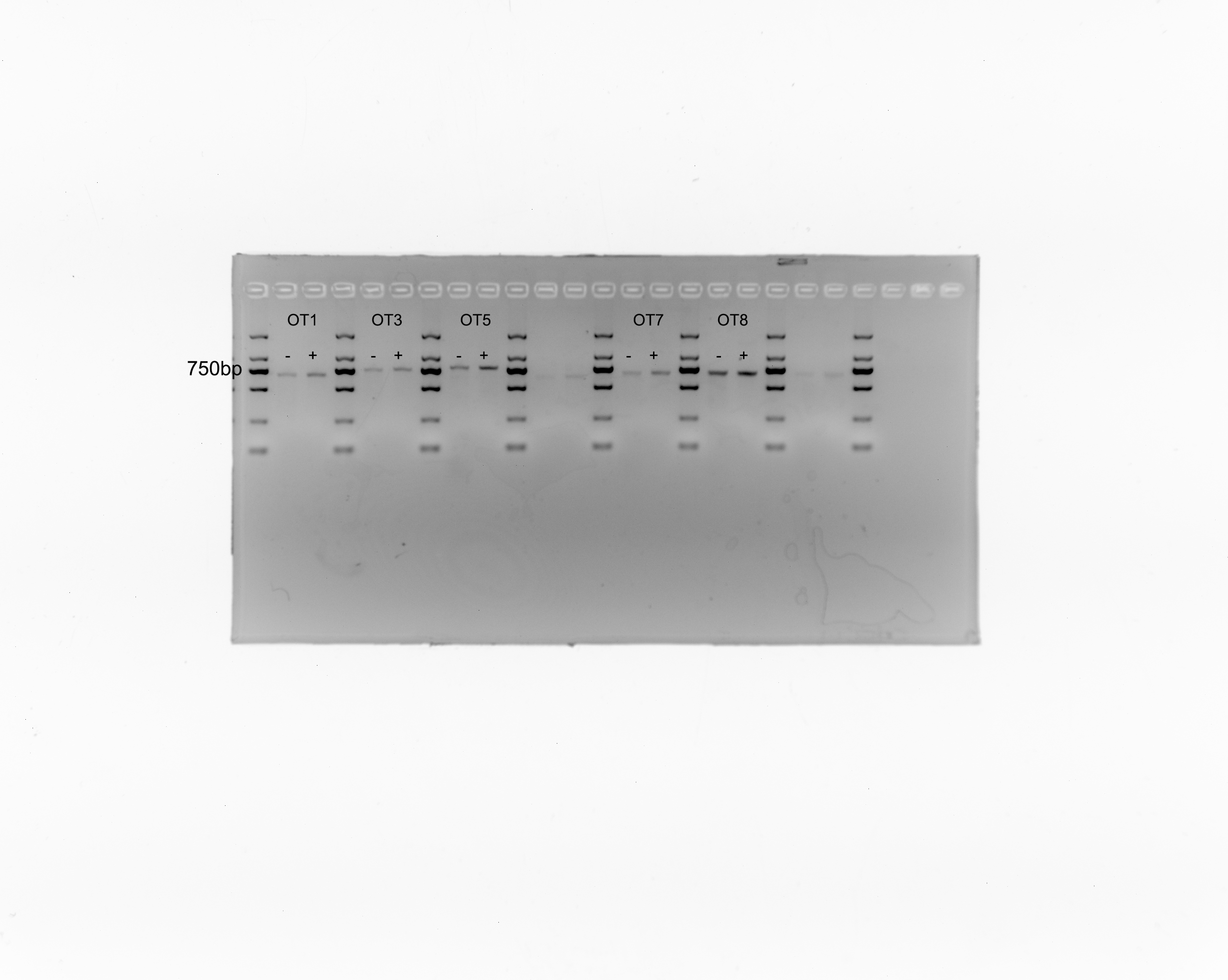

Supplement: Source data 2. [file elife-84065-data2.zip › Figure-Source Data 2/Figure 10-figure supplement 1-Source Data/Figure 10-figure supplement 1-Source Data1.jpg]

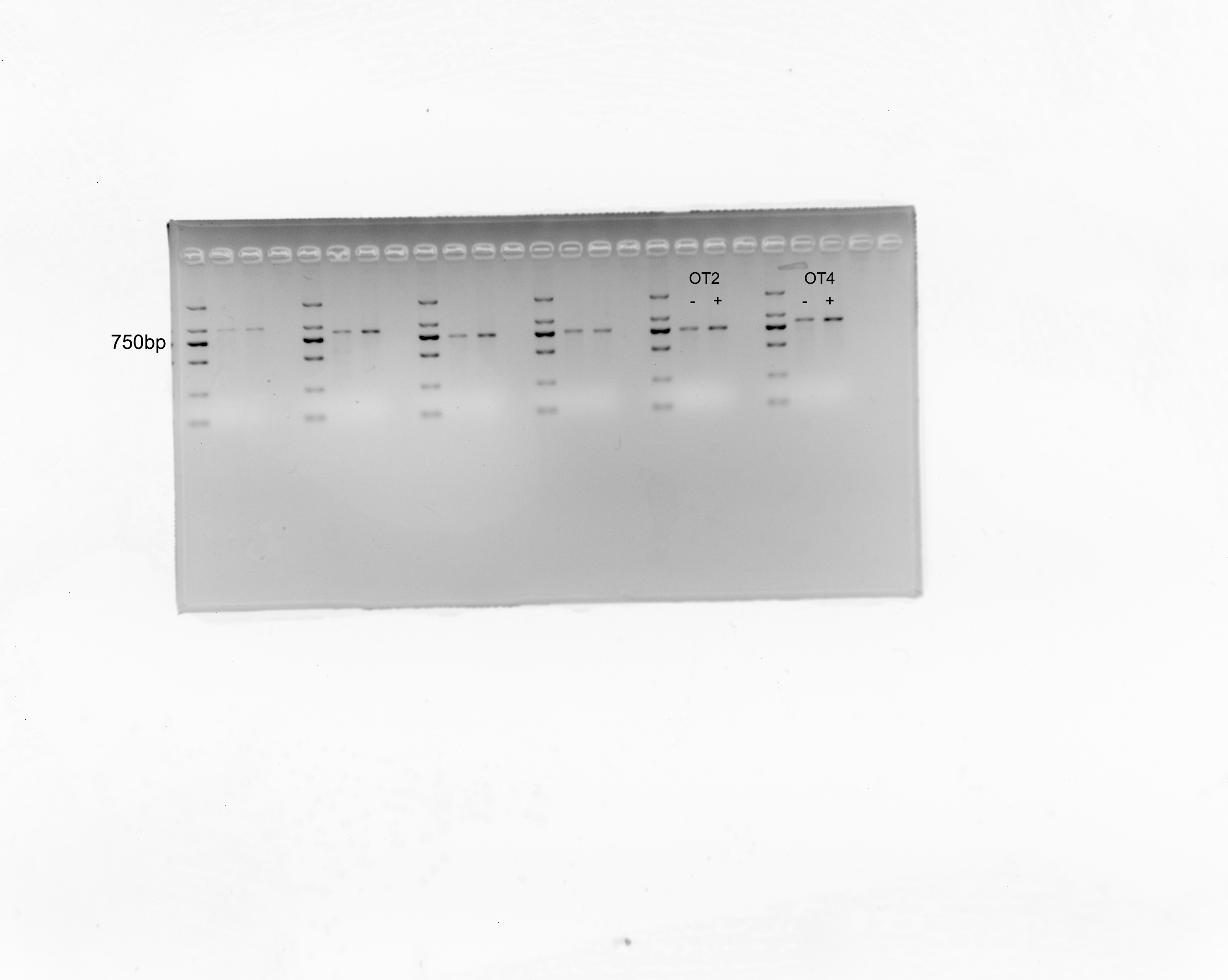

Supplement: Source data 2. [file elife-84065-data2.zip › Figure-Source Data 2/Figure 10-figure supplement 1-Source Data/Figure 10-figure supplement 1-Source Data2.jpg]

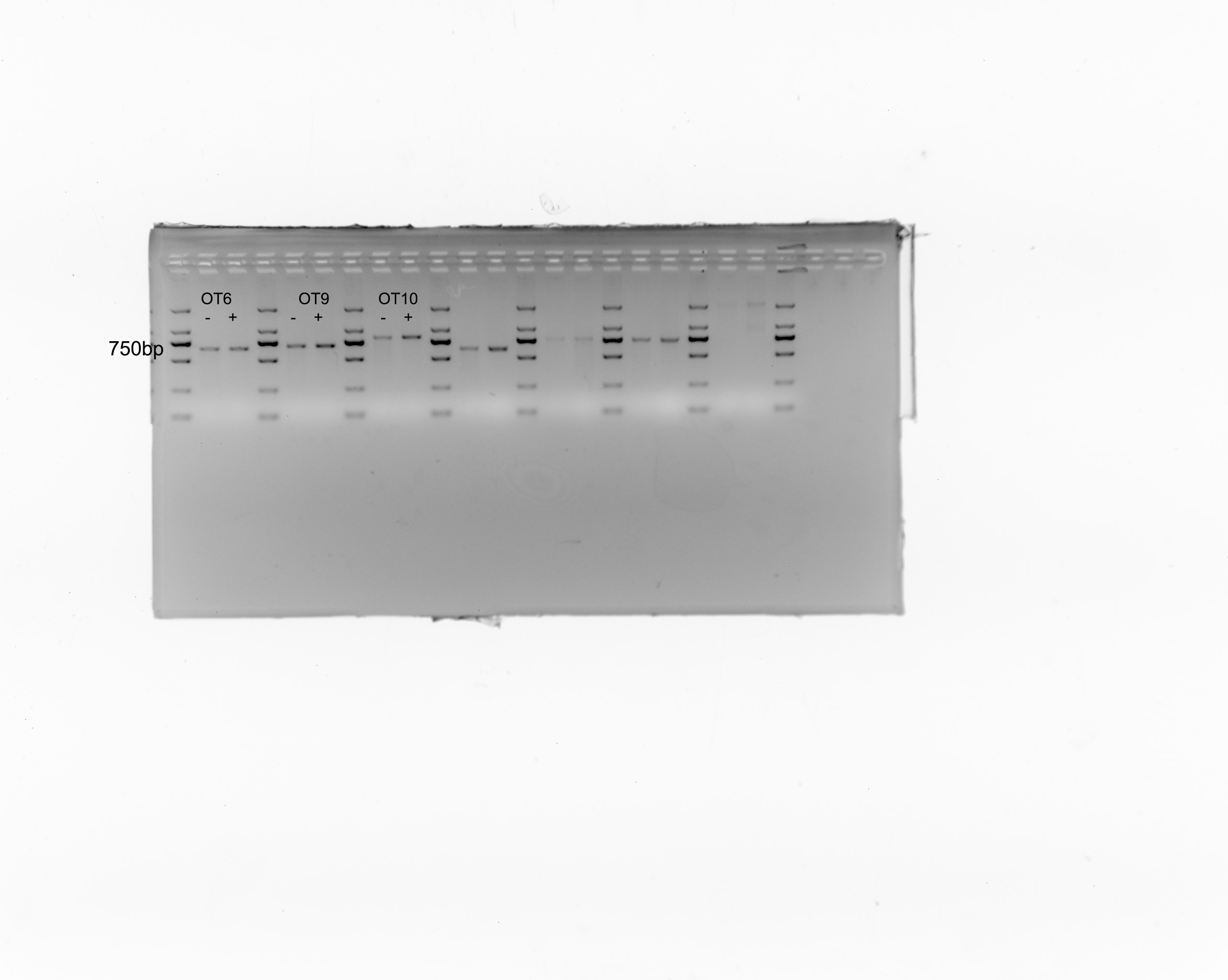

Supplement: Source data 2. [file elife-84065-data2.zip › Figure-Source Data 2/Figure 10-figure supplement 1-Source Data/Figure 10-figure supplement 1-Source Data3.jpg]

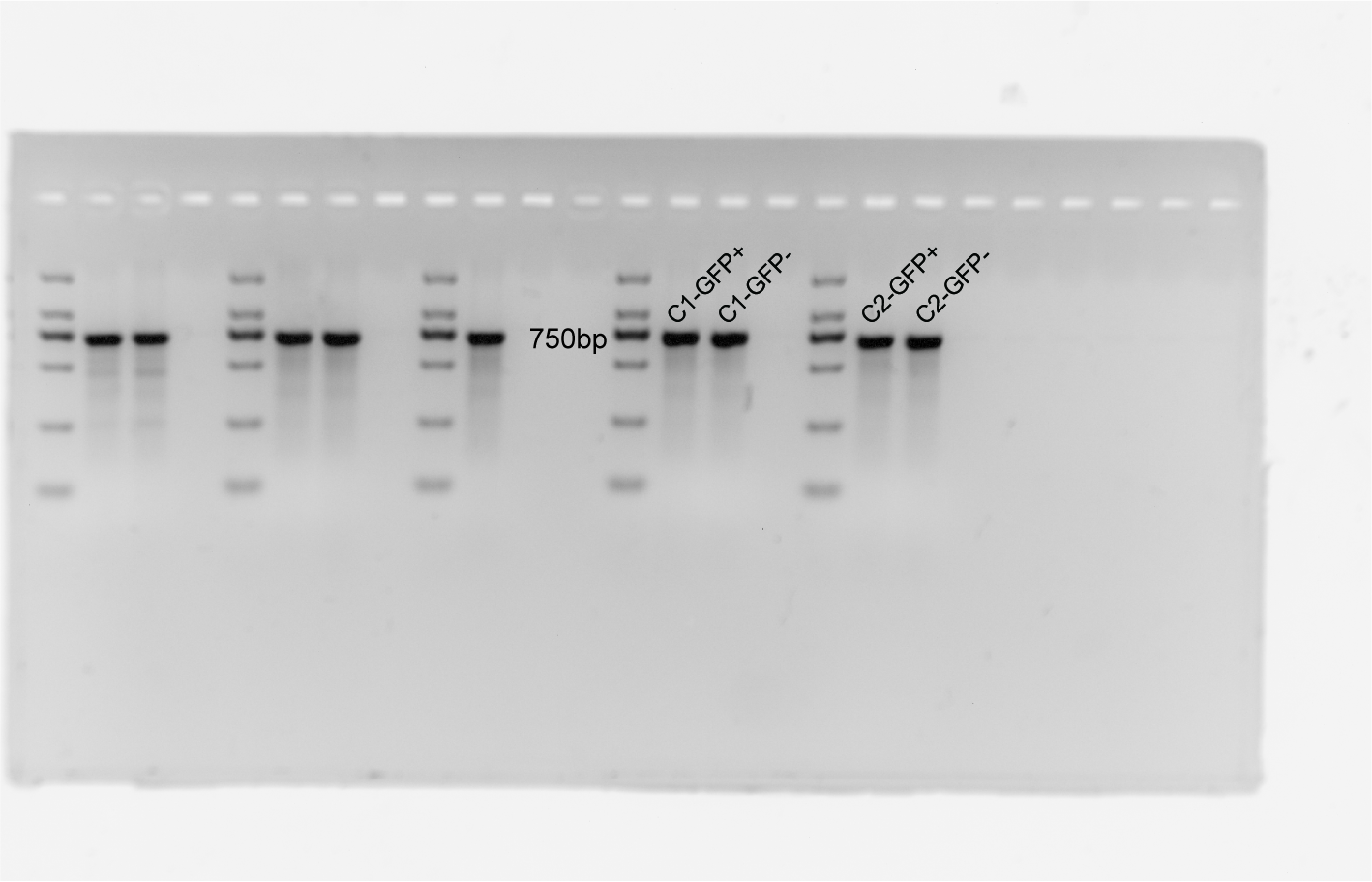

Supplement: Source data 2. [file elife-84065-data2.zip › Figure-Source Data 2/Figure 5-figure supplement 1-Source Data/Figure 5-figure supplement 1-Source Data1.tif]

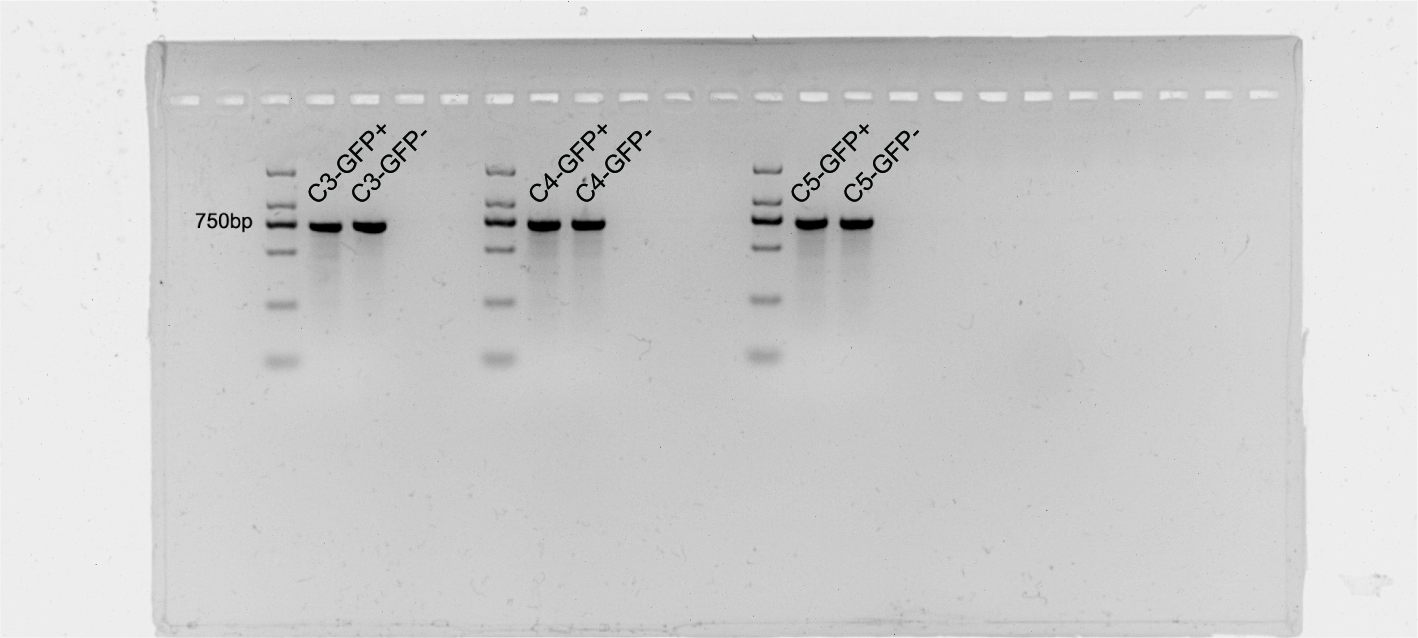

Supplement: Source data 2. [file elife-84065-data2.zip › Figure-Source Data 2/Figure 5-figure supplement 1-Source Data/Figure 5-figure supplement 1-Source Data2.tif]

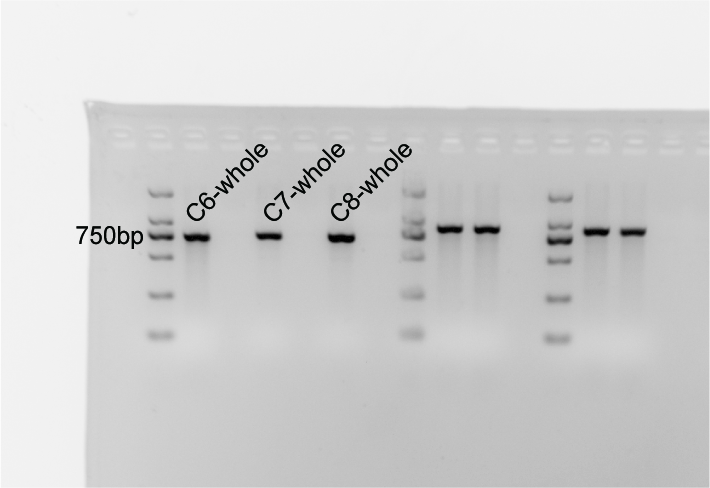

Supplement: Source data 2. [file elife-84065-data2.zip › Figure-Source Data 2/Figure 5-figure supplement 1-Source Data/Figure 5-figure supplement 1-Source Data3.tif]

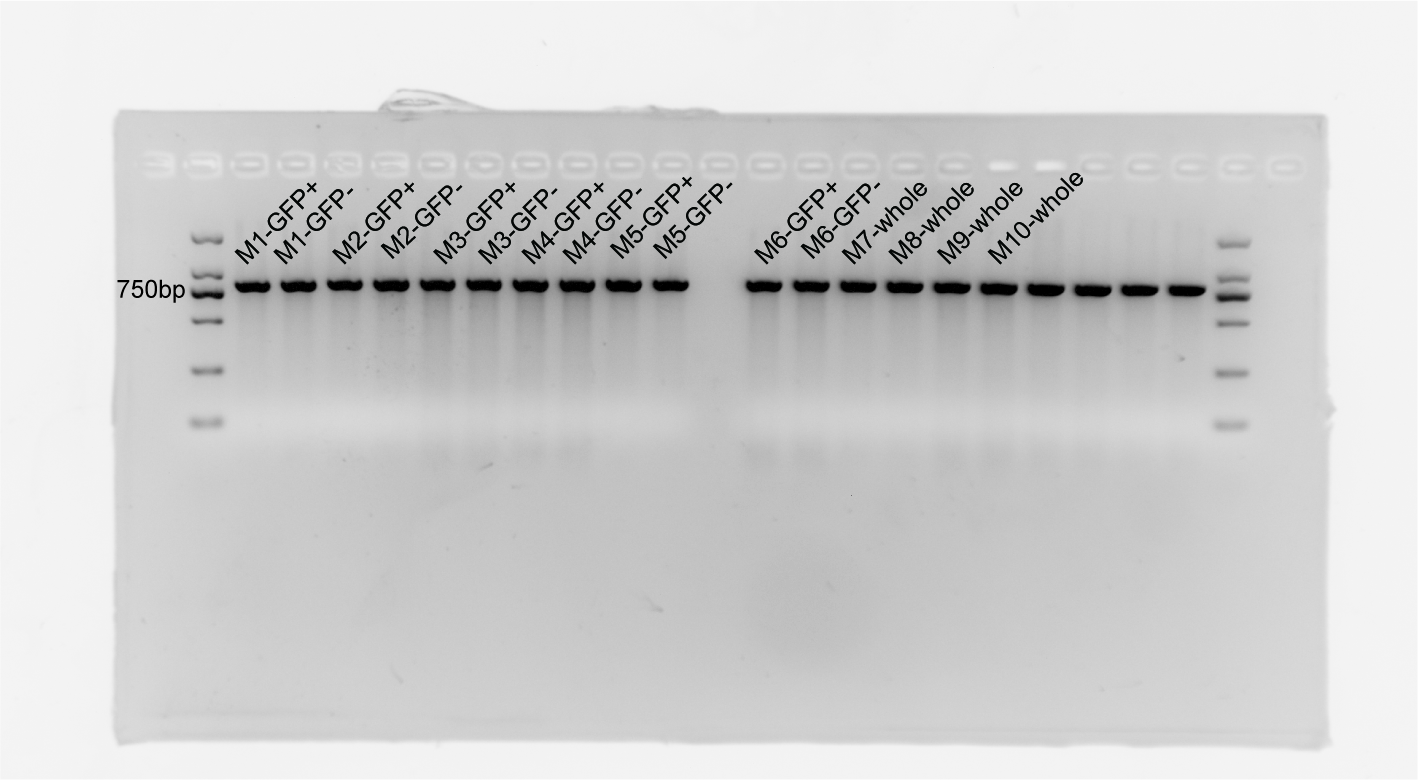

Supplement: Source data 2. [file elife-84065-data2.zip › Figure-Source Data 2/Figure 5-figure supplement 1-Source Data/Figure 5-figure supplement 1-Source Data4.tif]

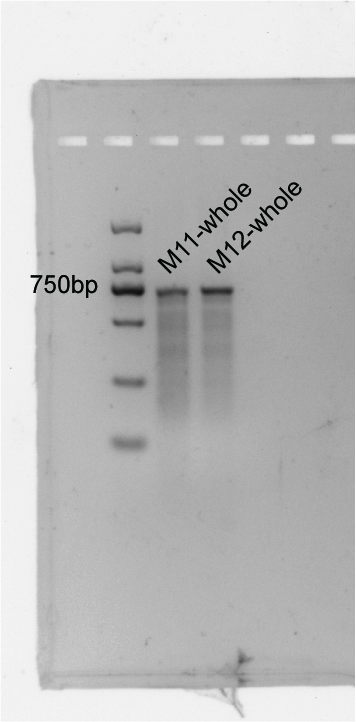

Supplement: Source data 2. [file elife-84065-data2.zip › Figure-Source Data 2/Figure 5-figure supplement 1-Source Data/Figure 5-figure supplement 1-Source Data5.tif]

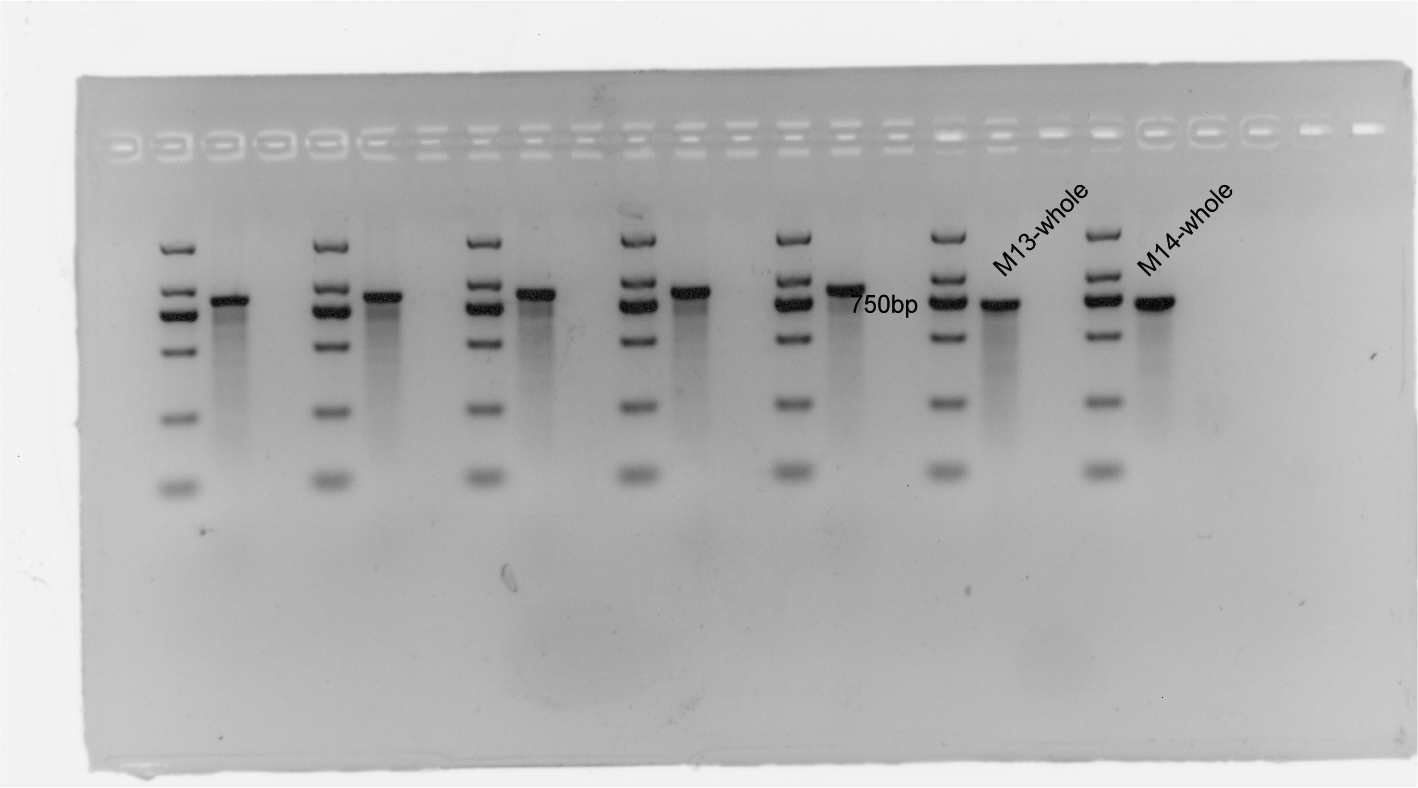

Supplement: Source data 2. [file elife-84065-data2.zip › Figure-Source Data 2/Figure 5-figure supplement 1-Source Data/Figure 5-figure supplement 1-Source Data6.tif]

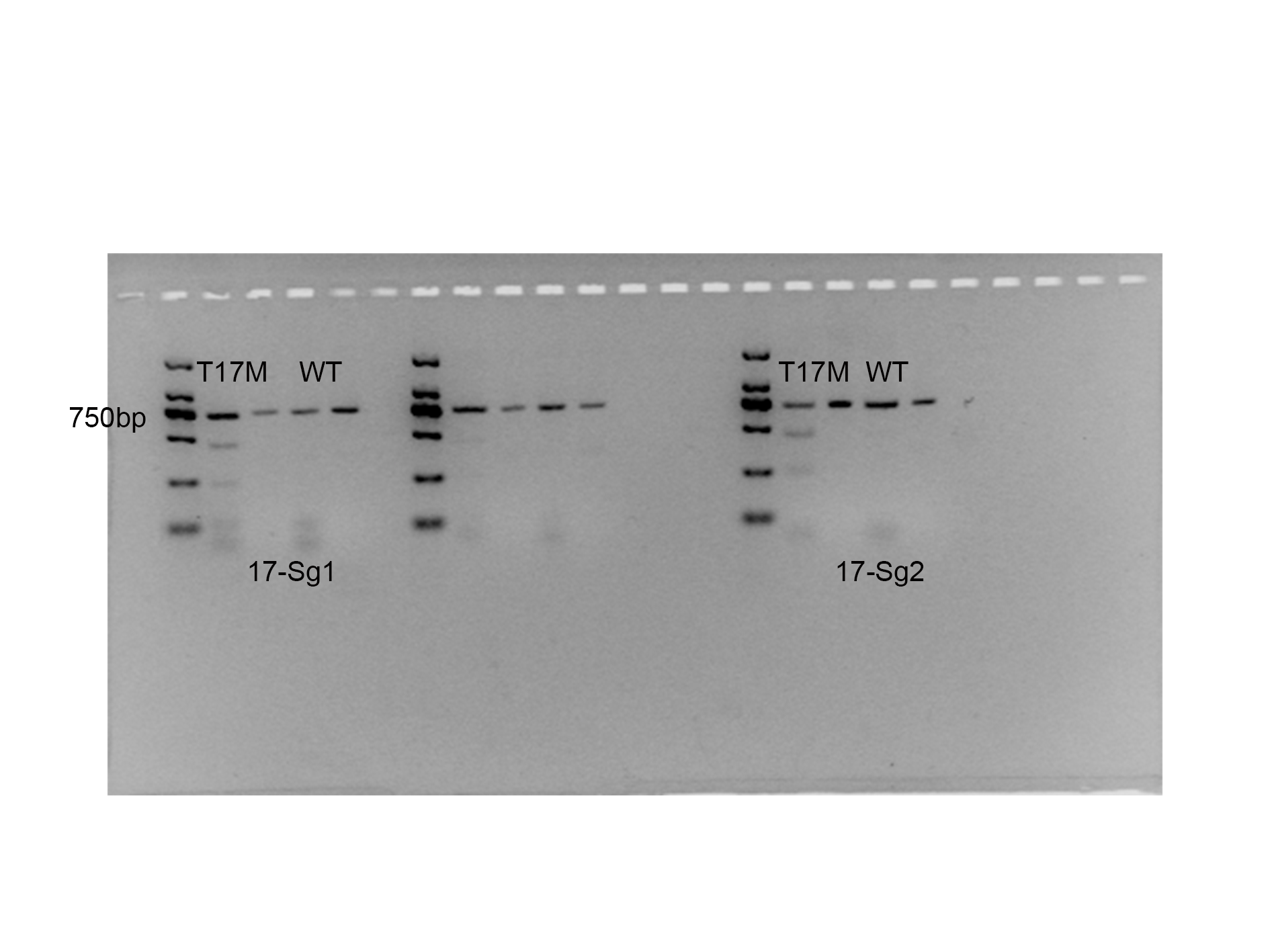

Supplement: Source data 2. [file elife-84065-data2.zip › Figure-Source Data 2/Figure1-Source Data/Figure 1-Source Data1.tif]

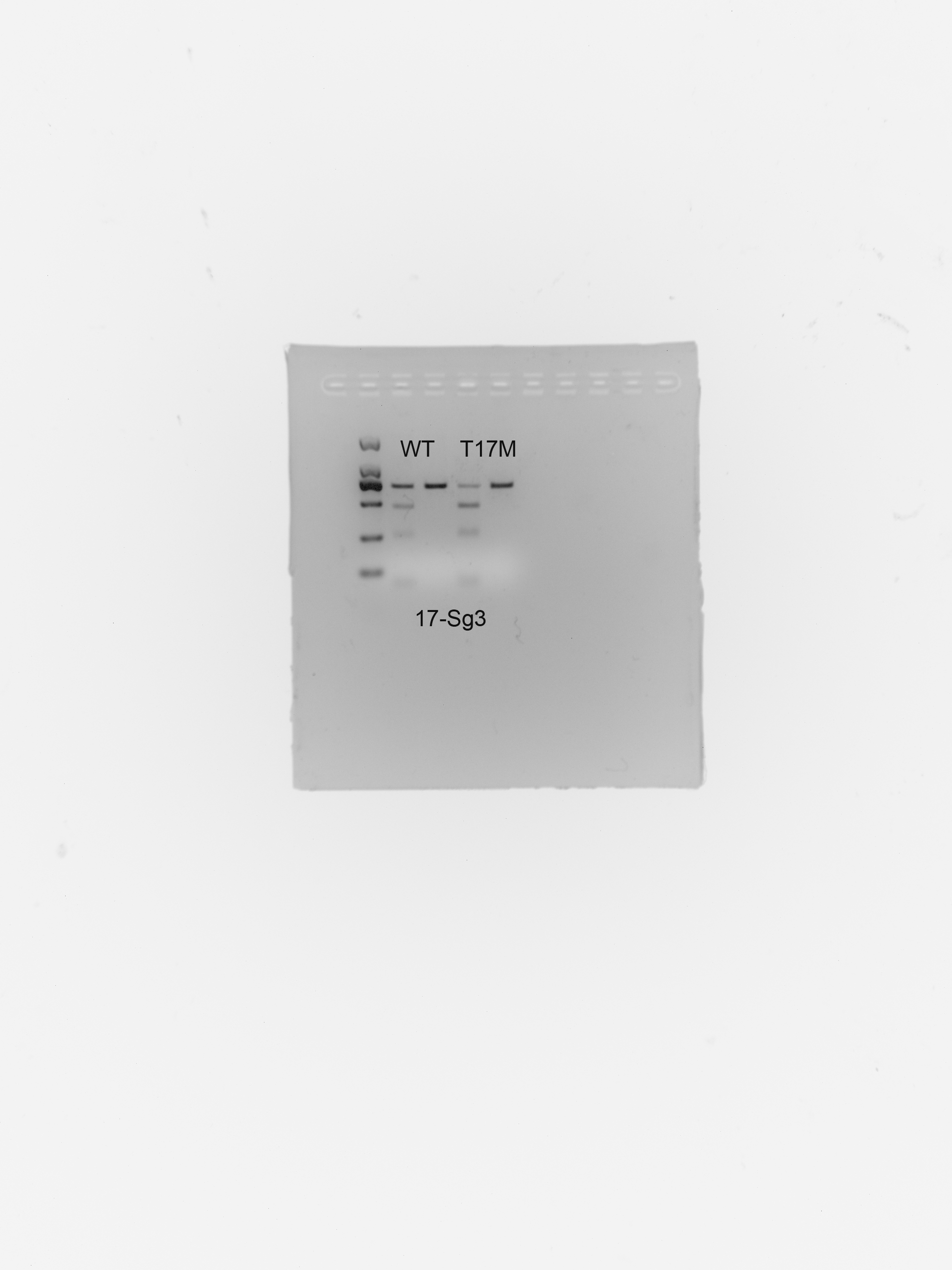

Supplement: Source data 2. [file elife-84065-data2.zip › Figure-Source Data 2/Figure1-Source Data/Figure 1-Source Data2.tif]

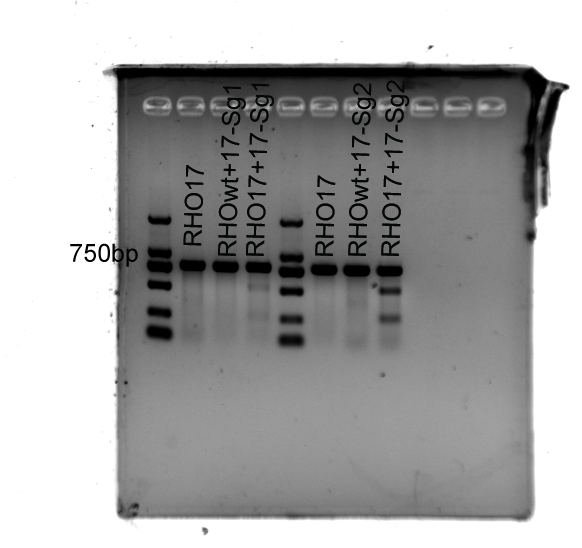

Supplement: Source data 2. [file elife-84065-data2.zip › Figure-Source Data 2/Figure2-Source Data/Figure 2-Source Data1.tif]

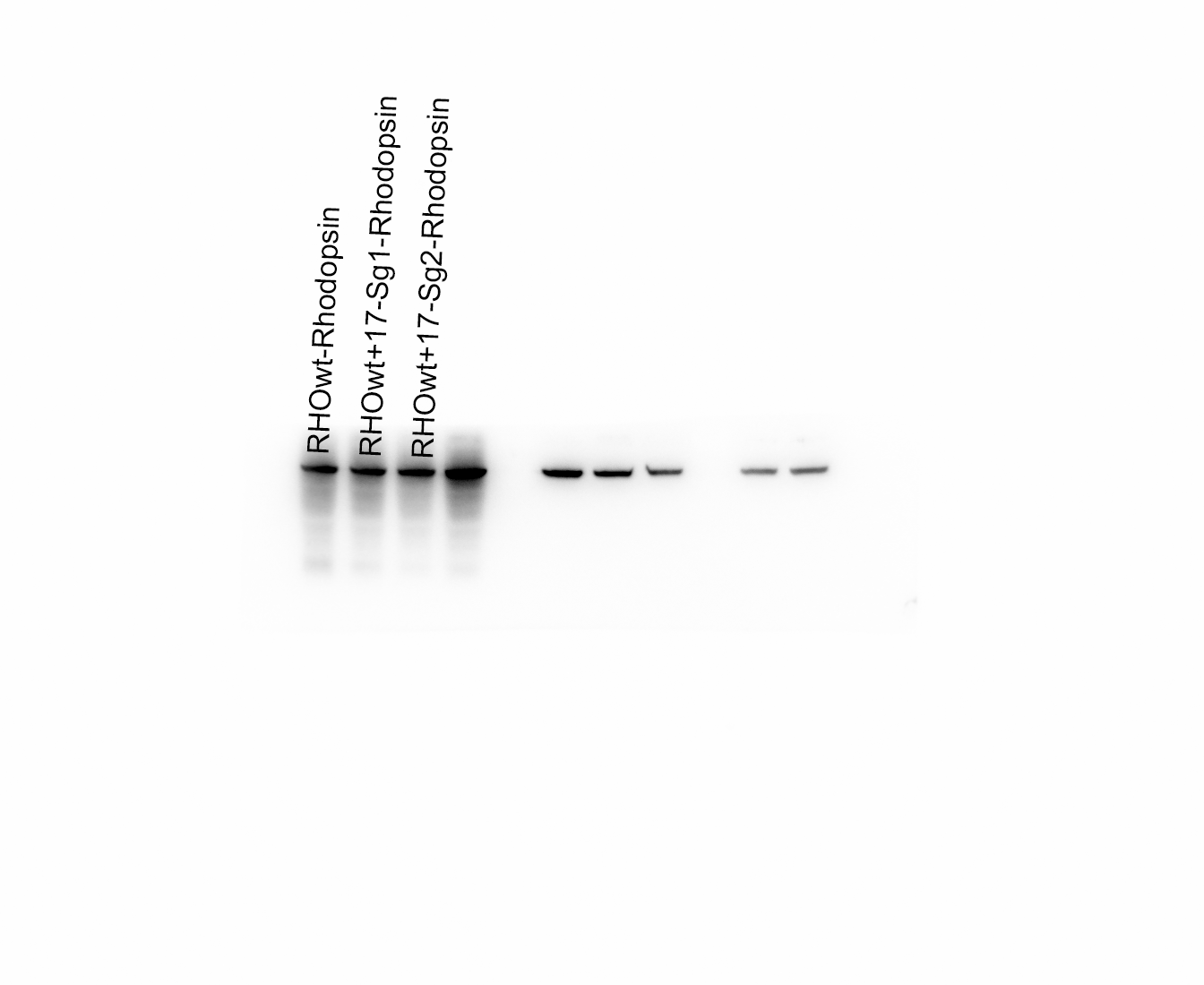

Supplement: Source data 2. [file elife-84065-data2.zip › Figure-Source Data 2/Figure2-Source Data/Figure 2-Source Data2.tif]

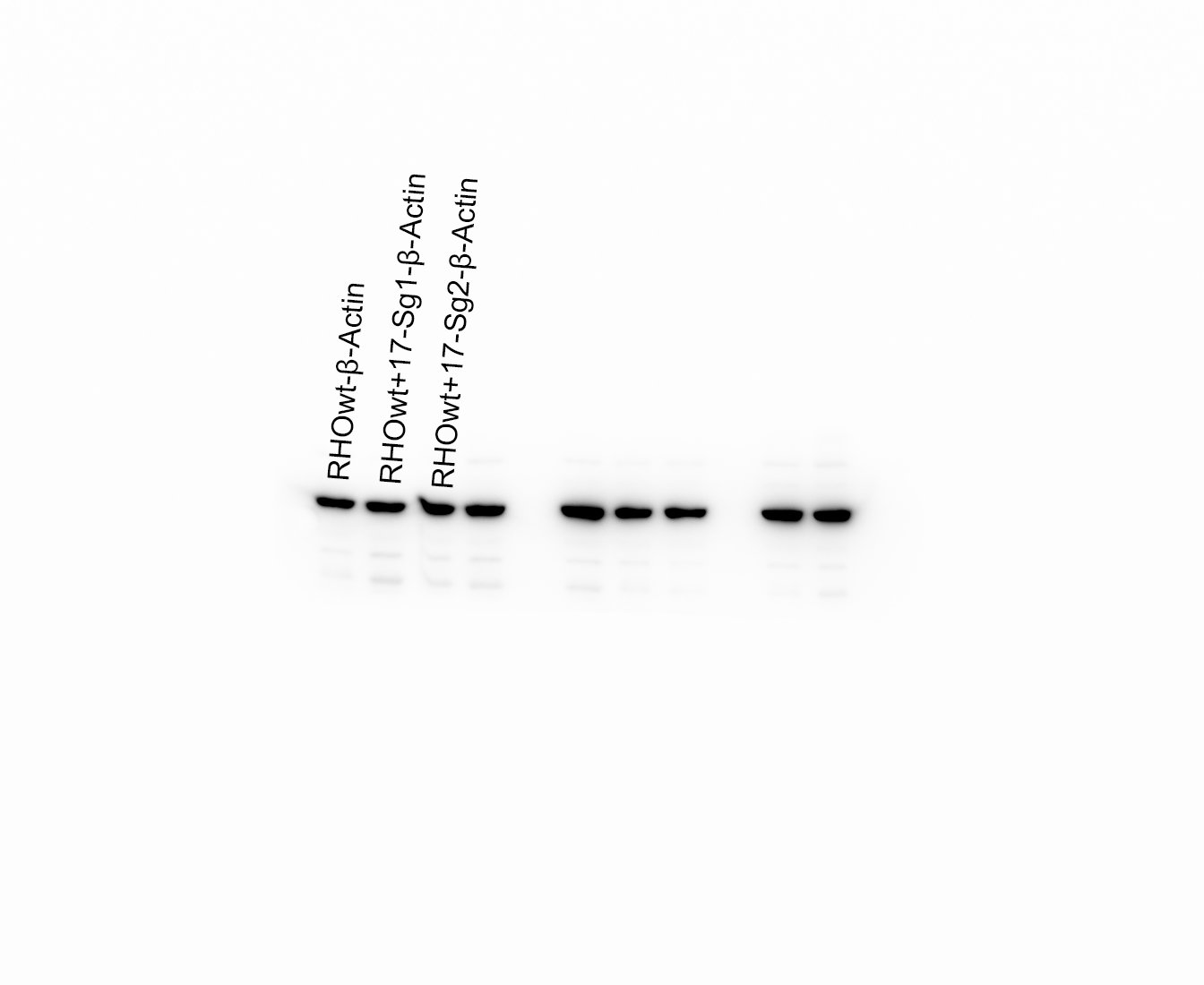

Supplement: Source data 2. [file elife-84065-data2.zip › Figure-Source Data 2/Figure2-Source Data/Figure 2-Source Data3.tif]

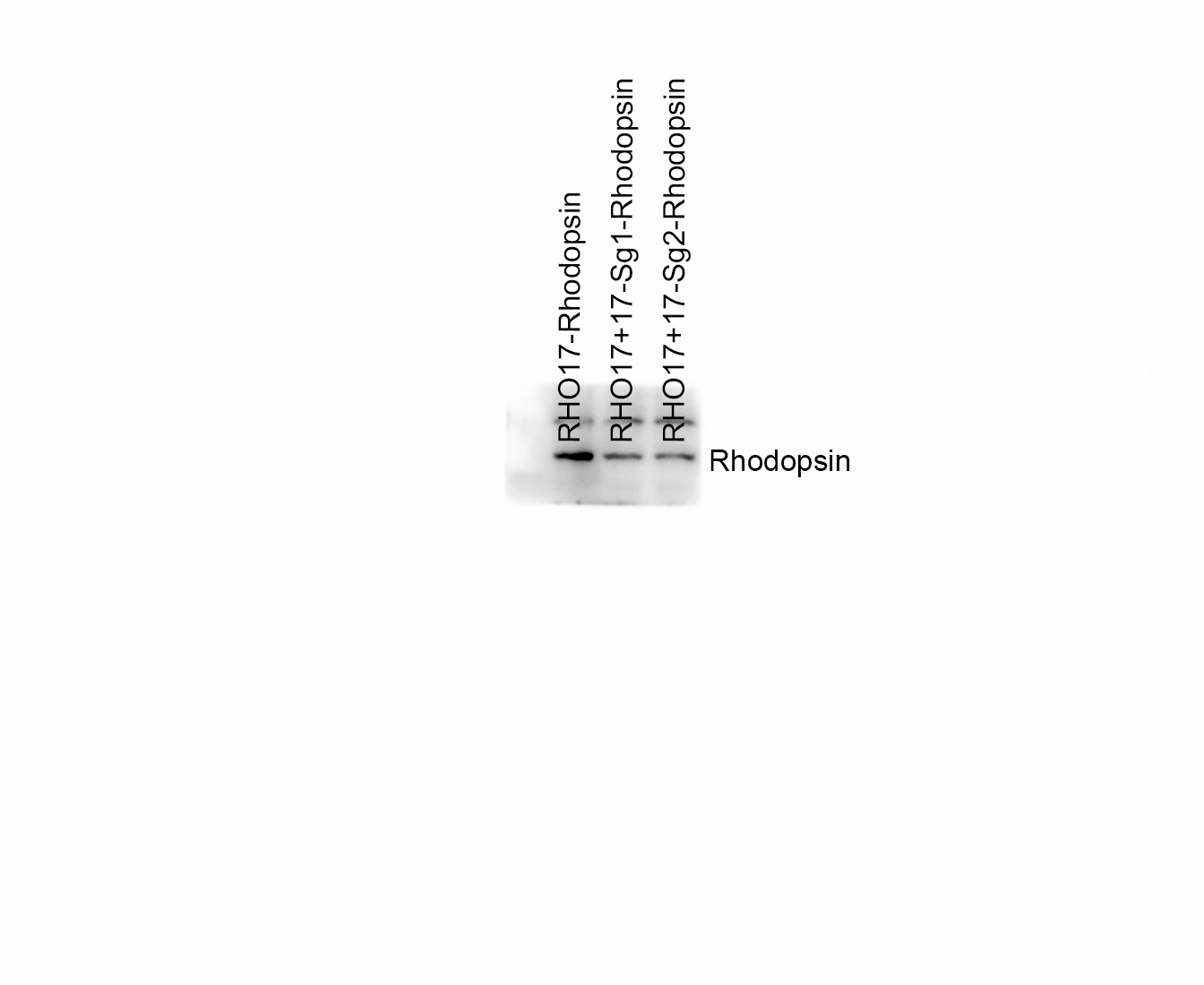

Supplement: Source data 2. [file elife-84065-data2.zip › Figure-Source Data 2/Figure2-Source Data/Figure 2-Source Data4.tif]

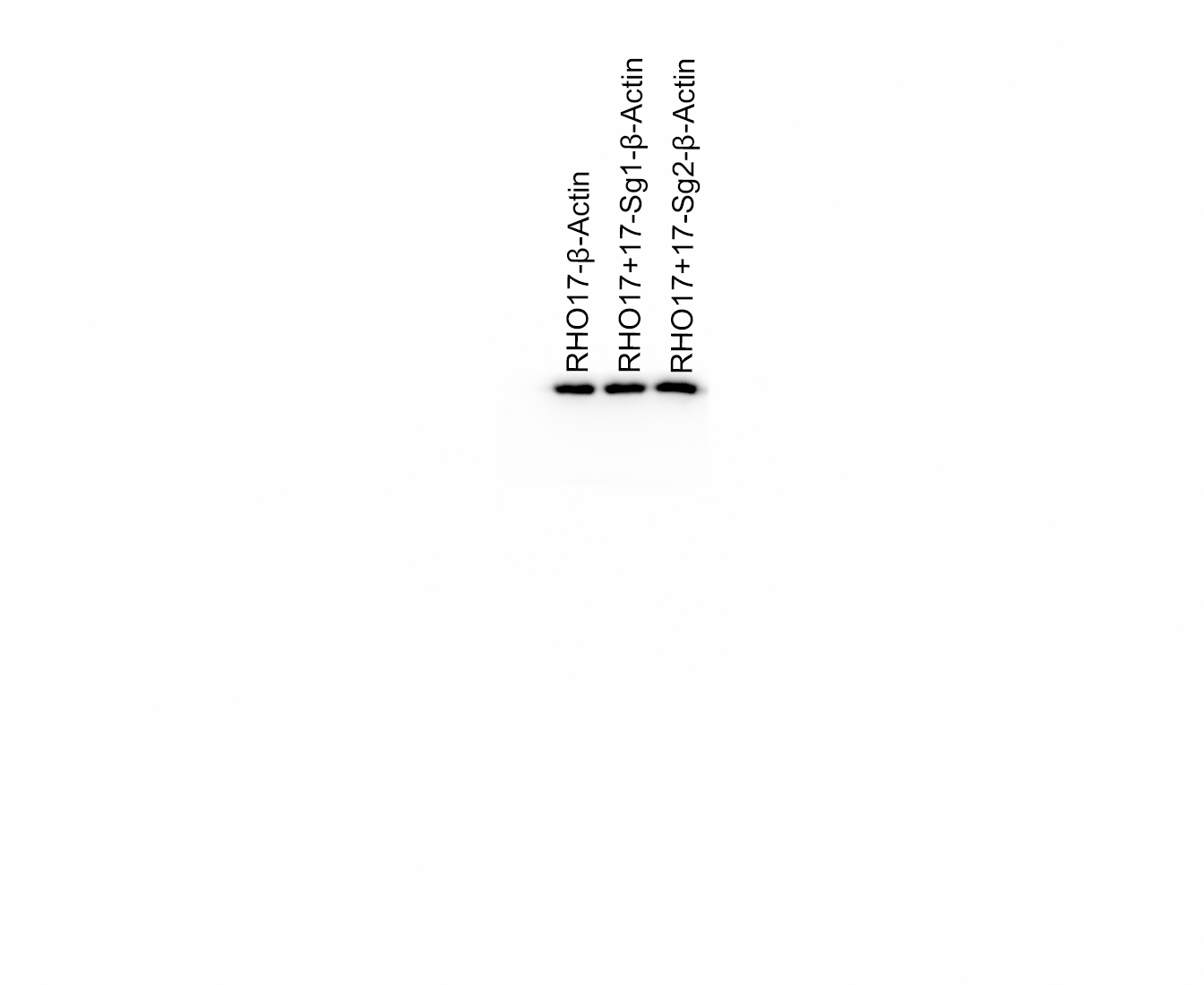

Supplement: Source data 2. [file elife-84065-data2.zip › Figure-Source Data 2/Figure2-Source Data/Figure 2-Source Data5.tif]

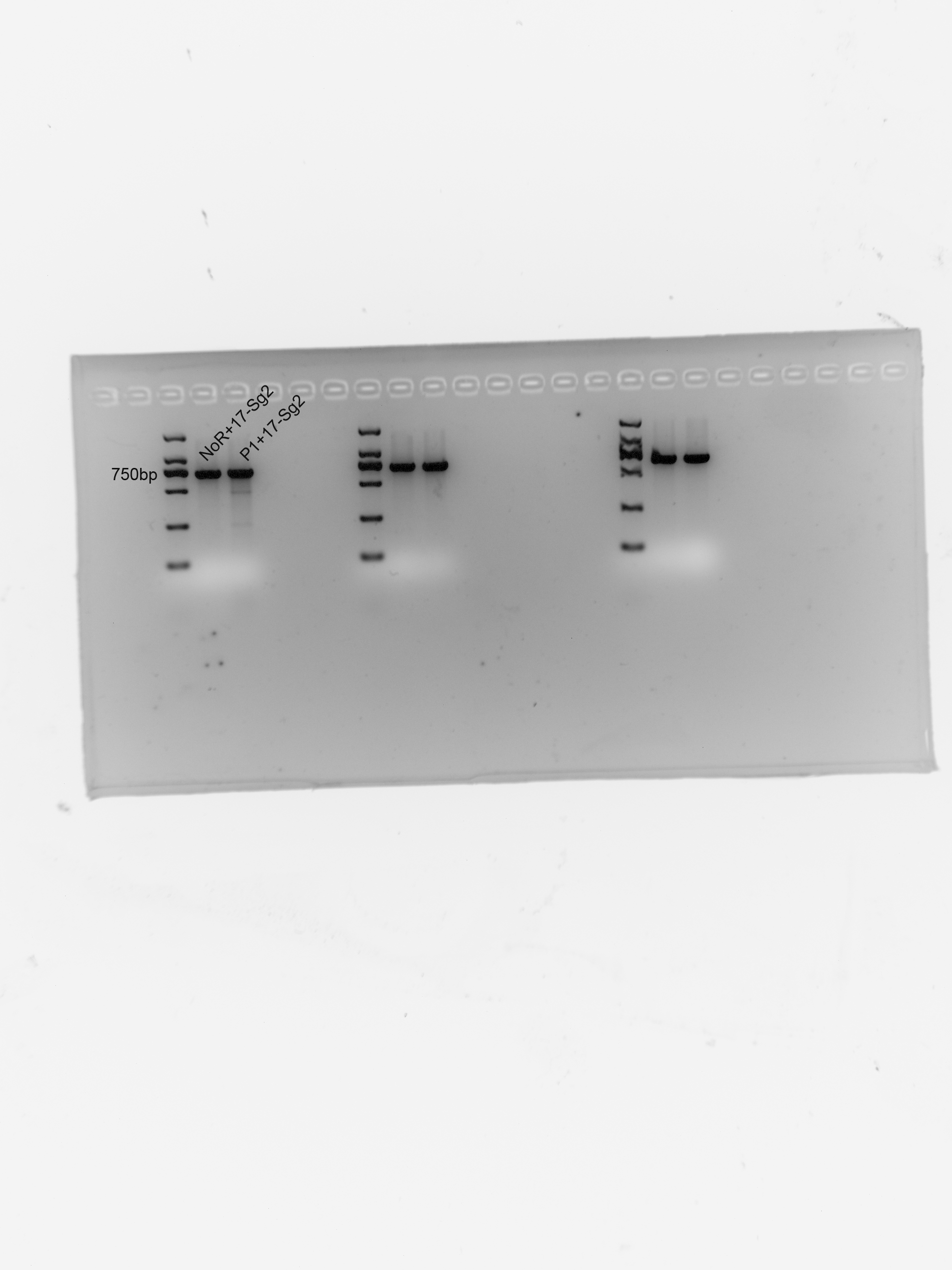

Supplement: Source data 2. [file elife-84065-data2.zip › Figure-Source Data 2/Figure3-Source Data/Figure 3-Source Data1.tif]

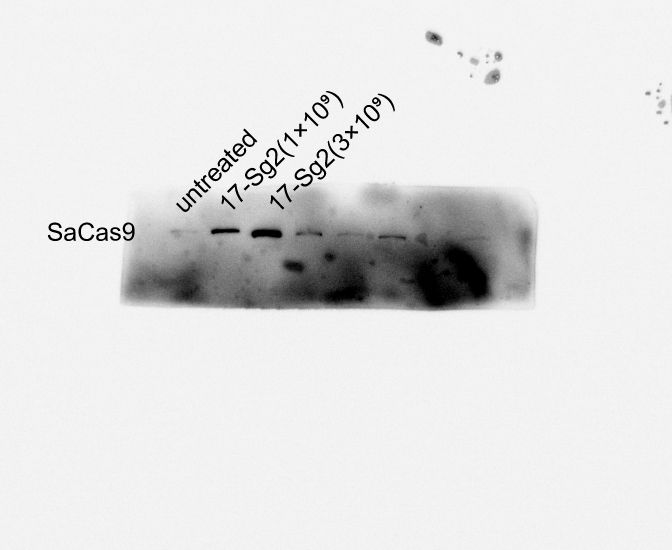

Supplement: Source data 2. [file elife-84065-data2.zip › Figure-Source Data 2/Figure4-Source Data/Figure4-Source Data1.tif]

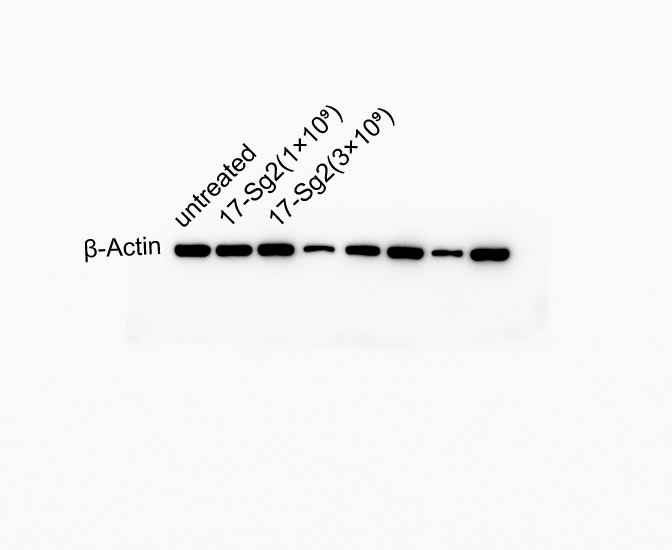

Supplement: Source data 2. [file elife-84065-data2.zip › Figure-Source Data 2/Figure4-Source Data/Figure4-Source Data2.tif]

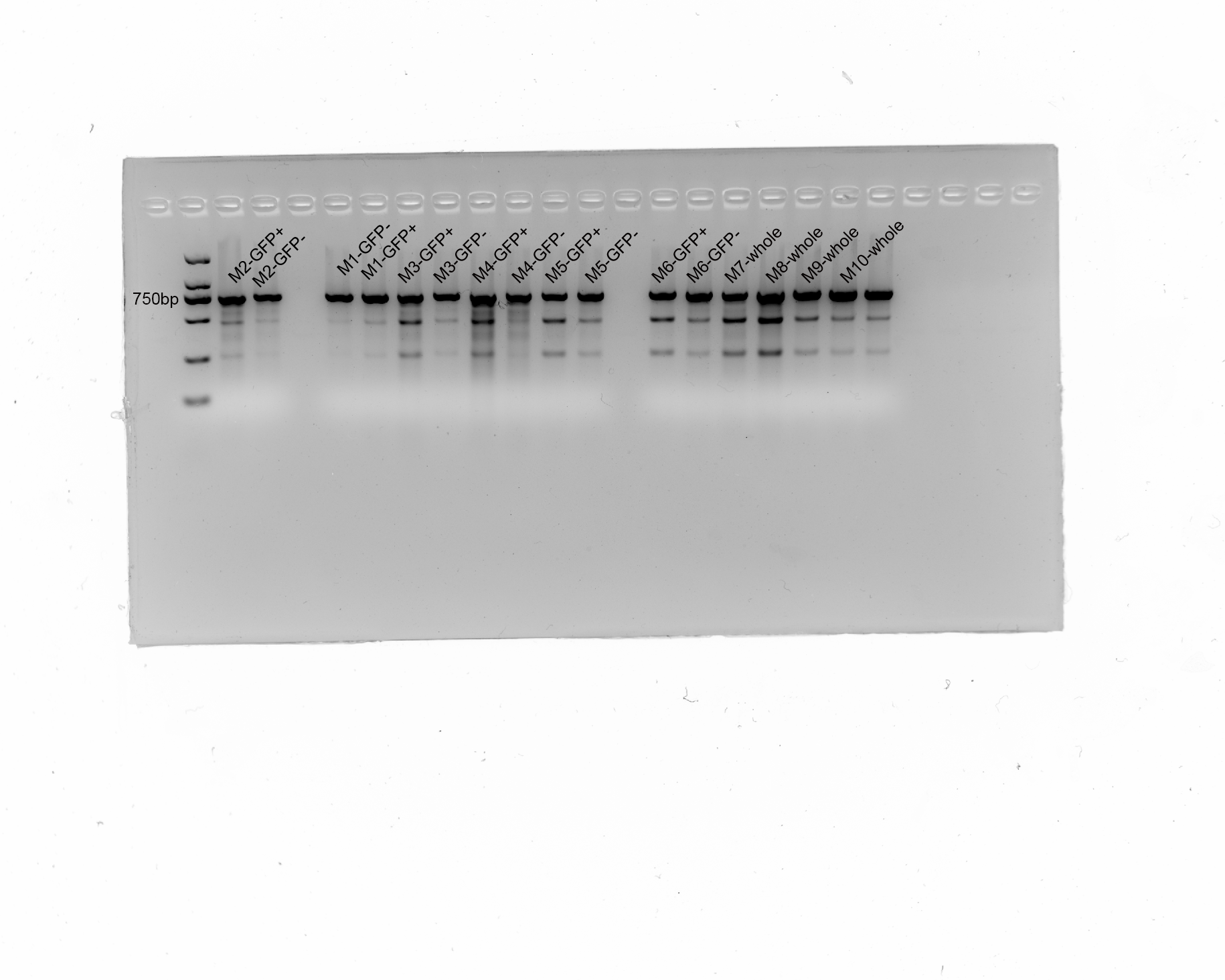

Supplement: Source data 2. [file elife-84065-data2.zip › Figure-Source Data 2/Figure5-Source Data/Figure 5-Source Data1.tif]

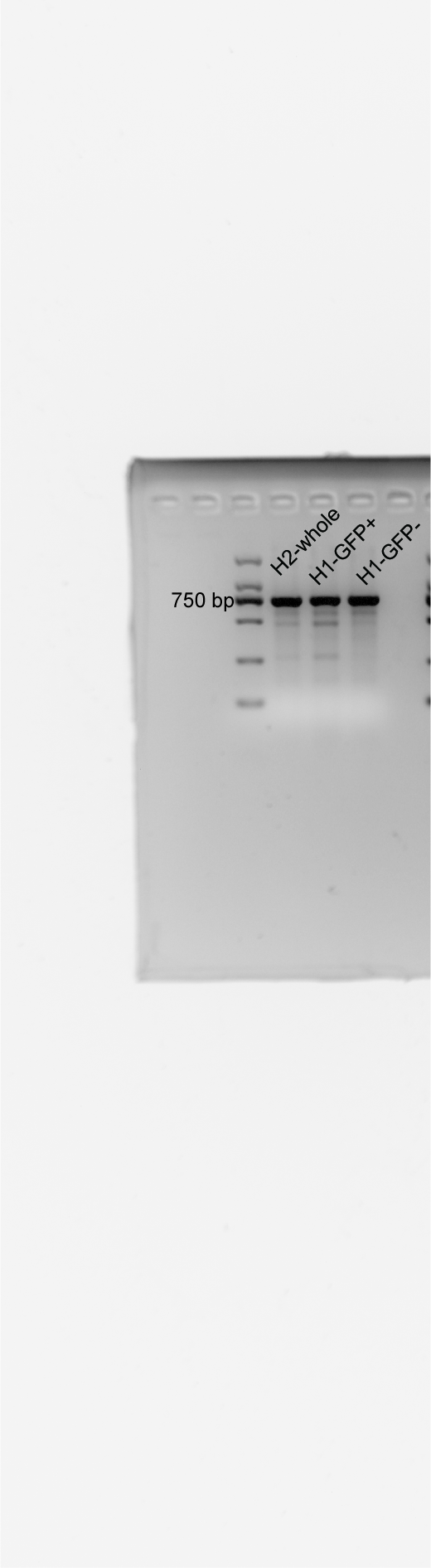

Supplement: Source data 2. [file elife-84065-data2.zip › Figure-Source Data 2/Figure7-Source Data/Figure 7-Source Data1.tif]

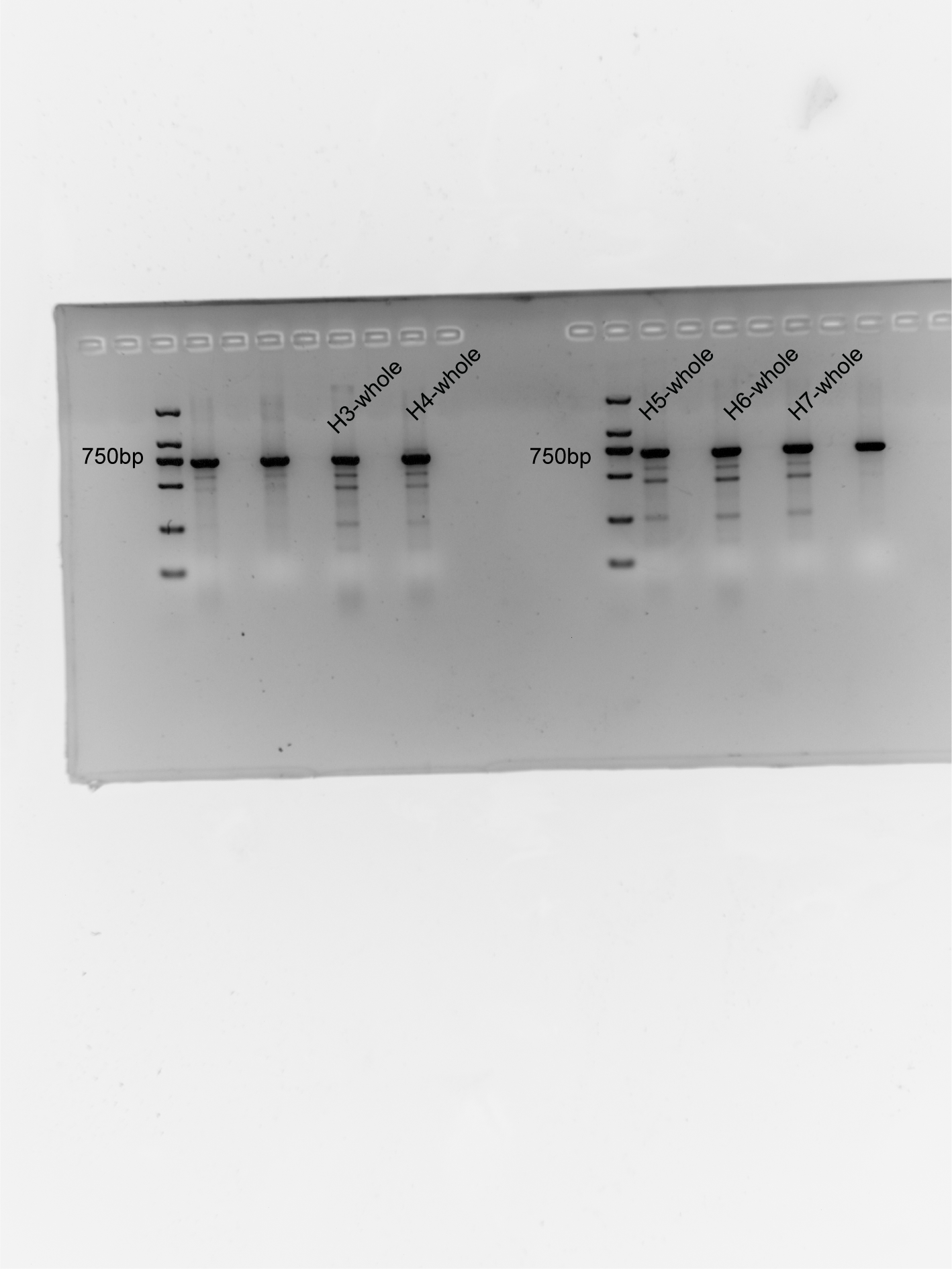

Supplement: Source data 2. [file elife-84065-data2.zip › Figure-Source Data 2/Figure7-Source Data/Figure 7-Source Data2.tif]

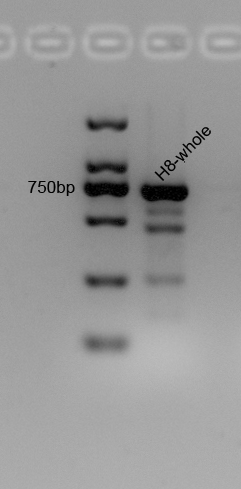

Supplement: Source data 2. [file elife-84065-data2.zip › Figure-Source Data 2/Figure7-Source Data/Figure 7-Source Data3.tif]
